# Survey of Recent Advances in Molecular Fluorophores, Unconjugated Polymers, and Emerging Functional Materials Designed for Electrofluorochromic Use

**DOI:** 10.3390/molecules28073225

**Published:** 2023-04-04

**Authors:** Ilies Seddiki, Brelotte Idriss N’Diaye, W. G. Skene

**Affiliations:** Laboratoire de Caractérisation Photophysique des Matériaux Conjugués Département de Chimie, Campus MIL, Université de Montréal, CP 6128, Succ. Centre-Ville, Montreal, QC H3C 3J7, Canada

**Keywords:** electrofluorochromism, reversible fluorescence turn off/on, electrochemically mediated fluorescence modulation, electrofluorochromic devices

## Abstract

In this review, recent advances that exploit the intrinsic emission of organic materials for reversibly modulating their intensity with applied potential are surveyed. Key design strategies that have been adopted during the past five years for developing such electrofluorochromic materials are presented, focusing on molecular fluorophores that are coupled with redox-active moieties, intrinsically electroactive molecular fluorophores, and unconjugated emissive organic polymers. The structural effects, main challenges, and strides toward addressing the limitations of emerging fluorescent materials that are electrochemically responsive are surveyed, along with how these can be adapted for their use in electrofluorochromic devices.

## 1. Overview

Electroluminochromism is reliant on an external light source to excite a broad class of intrinsic emitters whose emission intensity is subsequently modulated with an applied potential regardless of the excited-state manifold. The photoemission can either be quenched or enhanced with an applied potential. Modulating the photoexcited fluorescence occurring exclusively from the singlet excited state, such as with organic fluorophores, with an applied potential is defined as electrofluorochromism. The seminal electrofluorochromic works using a molecular fluorophore [1] and a conjugated fluorescent polymer [2] opened the field of modulating the photoemission of intrinsic emitters with applied potentials [3]. Despite these, electrofluorochromism, and the functional devices that exploit this property, are in their infancy compared to proven electrochromic (EC) devices that have been extensively evaluated [4,5,6]. EC devices reversibly change color with an applied potential. The electrochemically mediated changes that occur in the visible region have found uses in a range of consumer applications [7,8,9,10], including in devices for displays [4].

Due to the ever-increasing consumer demand for enhanced electronics, sensors, and detectors, electrofluorochromic (EFC) materials are warranting more attention than their simpler color-changing counterparts. Nevertheless, valuable insights have been gained from the color-changing organic functional materials used in EC devices, and these can be applied to operating EFC devices. It has been well established that the tuning of a material’s spectroscopic properties can be contingent on both its structure and composition in order to cover the entire visible spectrum [11,12,13,14]. Functional materials can also be made mechanically compliant with non-rigid electrodes, which paves the way for bendable, stretchable, and twistable EC devices [15,16,17,18,19,20,21]. They can be processed using a gamut of deposition techniques [22,23,24]. This opens the possibility of using them to coat substrates that span a range of sizes. Ultimately, this is advantageous in terms of upscaling the fabrication of electrochromic devices by large-scale-processing techniques such as roll-to-roll printing [24,25,26], spray coating [27,28], ink-jet printing [29,30,31], and slot-die coating [32].

Borrowing upon the knowledge of EC materials, EFC materials can be rationally designed to possess ideal properties for their use in the operation of EFC devices. The scope of this survey concerns the consolidation of the recent progress in the materials design of organic electrofluorochromic materials. Given the breadth of emissive materials for both electrofluorochromic [33,34,35,36,37,38,39] and electroluminochromic [40,41,42,43,44,45] applications that have been reviewed, advances made towards developing EFC materials that span the past five years are surveyed herein. Within this context, focus will be placed on materials whose emission is reversibly quenched with an applied potential according to three broad categories of electrofluorochromes: (1) molecular fluorophores coupled with redox-active moieties, (2) electroactive molecular fluorophores, and (3) unconjugated organic redox-active emissive polymers. These materials will be compared with limitedly representative examples of conjugated polymeric electrofluorochromes. While priority is given to materials’ EFC properties that have been investigated in devices, active materials that have also been evaluated in thin films and in solution will likewise be presented. General emission deactivation modes will also be surveyed to help the reader appreciate the fundamental photophysical processes of EFC materials and their structural requirements. These will be complemented with emerging materials that have the requisite properties for EFC use but have yet to reach their full EFC potential, along with key challenges in the field that have not yet been overcome.

## 2. Requisite Performance Criteria of Electrofluorochromes

Ideally, an EFC material should emit with a high fluorescence quantum yield (Φ_em_) when cast as a film. This expectation exists to ensure that photoemission variation with an applied potential can be visually detected in an operating solid-state device. Moreover, a large contrast ratio is desired for devices to be considered to have high performance. This is the ratio of the emission (on) and the quenched (off) states. The metric can also be expressed in % as the ratio of the difference between the emissive *on* and *off* states over the *on* state. However, intrinsic emitters typically only emit in dilute solutions and their emission is often quenched in the solid state. This arises from efficient intermolecular deactivation and inner filter effects [46,47]. Aggregation-induced emitters (vide infra) have the advantage of exhibiting the reverse effect [48,49]. Their emission is enhanced in the solid state owing to the suppression of otherwise excited-state deactivation modes. Therefore, EFC materials are evaluated in different states, such as in solutions, powders, and thin films, to assess their ultimate usefulness as the emitting material in functioning EFC devices.

**Figure 1:**
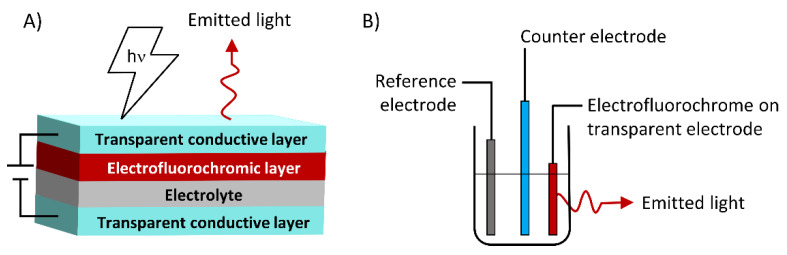
Different types of EFC devices used to evaluate the performance of EFC materials: (**A**) full device and (**B**) half-device.

Full and half-devices are the common types of devices used for evaluating the performance of electrofluorochromes. In a full device, the EFC material is sandwiched between two transparent electrodes along with an electrolytic gel. In the half-device, the EFC material is coated on a transparent electrode and the substrate is submerged in an electrolyte solution. In both configurations, the electrolyte is required to maintain electroneutrality once the EFC undergoes an electrochemical redox process. With regard to the full device (Figure 1), the gel consists of a polymer and a salt [33]. It is important for the polymer gel to be a transparent solid in which the salt is homogeneously dispersed in large concentrations to render the gel conductive. Another performance requirement of the polymer electrolyte matrix is that it must not dissolve the EFC material. Otherwise, the polymer electrolyte delaminates the emissive layer from the electrode, resulting in inconsistent emission and variable photoexcited emission over time. Similarly, the solvent used in half-devices must not dissolve the EFC material. Polymers are advantageous with respect to the evaluation of half-devices because their solubility is significantly reduced in solvents that are commonly used for electrofluorochromic measurements compared to their molecular counterparts. Therefore, they resist delamination from the electrode during multiple redox cycles with switching the applied potential. A poor solvent is judiciously chosen as the electrolytic solvent for half-devices to avoid dissolving the EFC material during the electrochemical operation. In contrast, their molecular counterparts are primarily evaluated in good solvents to ensure their complete solubility in the electrolytic solution. Suitable solvents include THF, dichloromethane, acetonitrile, and DMF because they can dissolve the organic electrofluorochrome. More importantly, these solvents dissolve the electrolytic salt in high concentrations and thus meet the conductivity requirements for their electrochemical use. Optically narrow (<2 mm) cuvettes are generally used for solution measurements. This choice is made to suppress diffusion of the EFC material and completely deplete its neutral state with an applied potential, resulting in a maximum emission contrast ratio.

An additional underlying criterion of the charge-balancing components is that they must not quench the intrinsic emission of the EFC material. This is significant due to the sensitivity of the excited-state fluorophores, which leads to undesired emission quenching when the device is not operating. The large effective concentration of the electrolyte in the full device can often degrade the emission of the EFC material during multiple redox switches. In contrast, the electrolyte is more dilute in the half-device, leading to colorfastness and the consistent emission of the EFC layer with multiple redox cycles. The electrolyte reversibly penetrates the light-emitting layer in both types of devices during their operation. Even when the employed device is in its resting state, the electrolyte remains embedded in the emissive layer after repeated redox cycles. This can reduce the amount of light emitted by the device when photoexcited. The desired degree of emission quenching can be mitigated by developing EFC materials that have high solid-state-emission quantum yields.

## 3. Fluorescence Deactivation Modes

**Figure 2:**
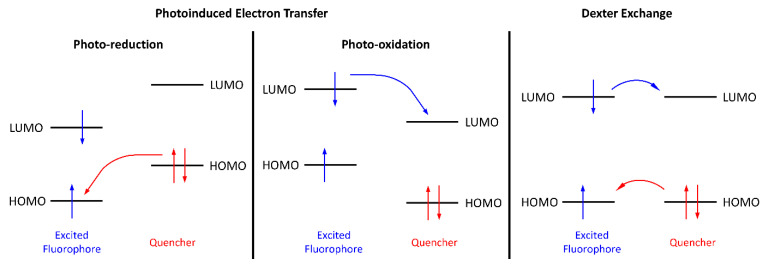
Photoinduced emission deactivation mechanisms: photoreduction (**left**) and photooxidation (**middle**) of the emitter and Dexter pseudo-electron exchange (**right**).

Electrofluorochromes are typically intrinsically fluorescent emitters that are π-conjugated aromatics. They have a high Φ_fl_ owing to their rigid structures that prevent unwanted emission deactivation by non-radiative modes such as bond rotations. By understanding intrinsic emission deactivation modes, emitters can be exploited to trigger redox-mediated photoemission intensity modulation. The variation in fluorescence with the applied potential can occur via several mechanisms. The most often-encountered emission-quenching mode of fluorophores, to which an electroactive group for EFC modulation is covalently attached, is photo-induced electron transfer (PET) [50]. This process can take place via one of two mechanisms, depending on the energy levels of the emitter and the electroactive component. The photoexcited fluorophore can be reduced by the electroactive specie when it is excited by light, according to Figure 2 (left panel). Emission quenching occurs via electron transfer from the deactivator’s HOMO to the corresponding level of the emitter, which is unfilled by the photoinduced promotion of an electron to the LUMO. This process is efficient when the HOMO energy levels of the deactivator and the emitter are respectively exergonic. In contrast, deactivation by photoinduced oxidation (Figure 2, middle panel) of the fluorophore occurs via exergonic electron transfer from its LUMO to the acceptor’s LUMO. The energy levels are modified with an applied potential, which leads to either the activation or deactivation of the PET mechanism. In the case of the photo-oxidation of the fluorophore by a covalently coupled redox-active moiety in a typical electrofluorochrome, the fluorescence-quenching mechanism can be suppressed by electrochemically reducing the redox-active component. Here, the energy levels of the reduced redox-active segment are perturbed, and the photoinduced electron from the fluorophore to the redox-active group is no longer exergonic. PET is subsequently deactivated, and the emission of the EFC material is turned on. Similarly, the photoreduction deactivation of the fluorophore can be suppressed by electrochemically oxidizing the redox-active moiety. The energy levels of the subsequently oxidized redox-active segment become endergonic, resulting in suppressed PET and the activation of fluorescence. In the absence of a quencher, the intrinsic emission of the emitter can similarly be suppressed with an applied potential (vide infra).

A similar electron transfer deactivation mode can occur when the electroactive moiety of the electrofluorochrome is electrochemically activated (vide infra). An electron-deficient radical cation, which is produced by electrochemical oxidation, can deactivate the emitter via photoinduced electron transfer from the fluorophore. This process occurs from the excited emitter to the singly occupied molecular orbital (SOMO) of the radical cation. Similarly, the electrochemical reduction of the electroactive segment can deactivate the emitter via electron transfer from its SOMO. 

The intrinsic emission of the fluorophore can additionally be perturbed by intramolecular charge transfer (ICT) between complementary electron-donating and -accepting moieties in the photoexcited state [51,52,53,54]. The charge transfer is promoted in polar environments, and it stabilizes the overall energy of the fluorophore. This reduces the HOMO–LUMO energy gap and affects fluorescence. The emission from the ICT state is red-shifted relative to the local excited state. Narrowing the energy gap increases the vibrational coupling between the HOMO and LUMO levels, resulting in fluorescence quenching. Overall, fluorophores that emit in the visible region have narrow energy gaps, resulting in reduced fluorescence quantum yields. The Φ_fl_ is further reduced in polar environments that favor intramolecular charge transfer. Therefore, emitters with high Φ_fl_ are desired to overcome unwanted inherent deactivation modes in assembled devices and afford devices that have high fluorescence contrast ratios. ICT can be perturbed to regulate photoinduced emission by electrochemically modulating the charge-transferring groups on the fluorophore. This makes it an ideal method for enabling electrofluorochromism.

**Figure 3:**
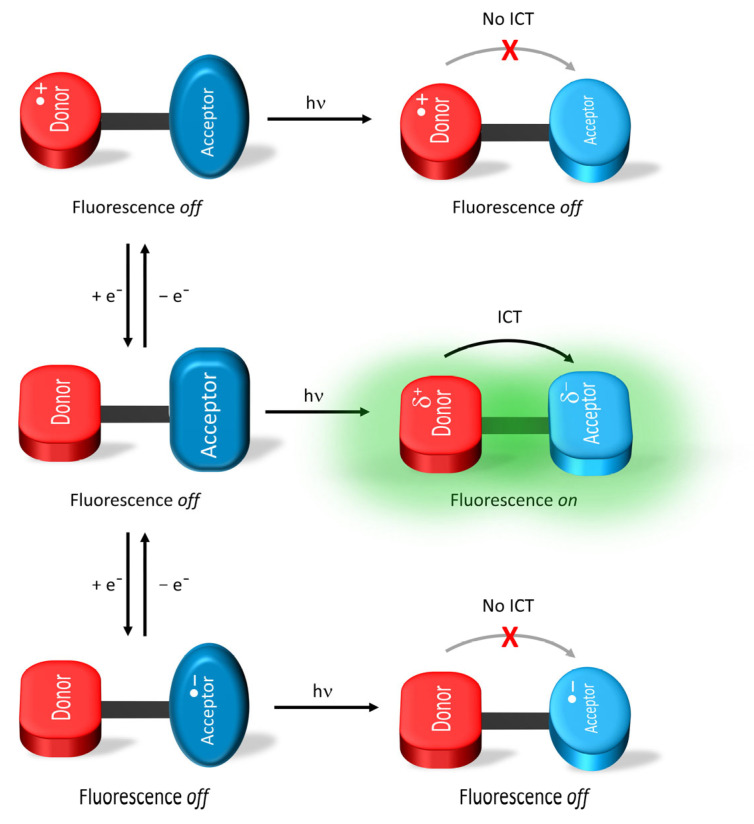
Electrofluorochromic mode of action of photoemission modulation by twisted intramolecular charge transfer.

A variation of ICT for florescence modulation is twisted intramolecular charge transfer (TICT). The electronic decoupling between a covalently attached donor and acceptor is perturbed by a change in the photoexcited conformation. The twisting of the otherwise near orthogonal donor–acceptor pair to planarity upon photoexcitation promotes electronic coupling and intramolecular charge transfer from the donor to the acceptor. Subsequently, the planar charge transfer state emits fluorescence and the donor–acceptor pair returns to its near-orthogonal ground state configuration (middle panel Figure 3). The donor–acceptor pair continues to twist and it adopts a coplanar configuration upon photoexcitation when either the acceptor is reduced (lower panel) or the donor is oxidized (top panel) electrochemically. However, the charge imbalance between the electrochemically modified donor–acceptor pair suppresses ICT, and no emission occurs. Suppressing the emissive TICT electrochemically has proven to be a viable mode of action for the production of EFC materials [55,56].

**Figure 4:**
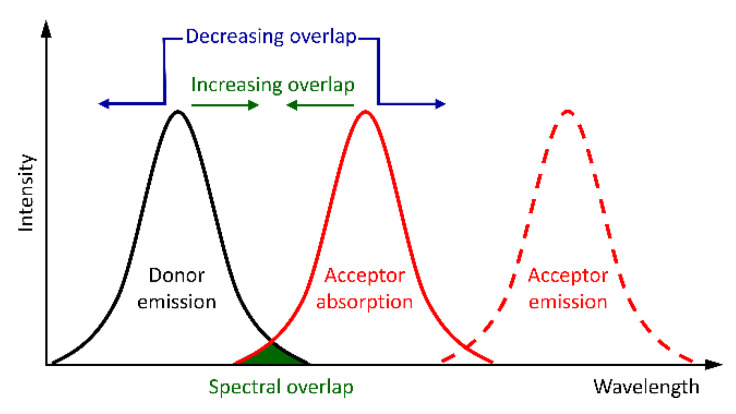
FRET activation and deactivation via electrochemically adjusting the spectral overlap integral for EFC use.

Non-radiative energy transfer of the EFC material can also occur according to two distinct mechanisms: Dexter pseudo-electron transfer and Forster resonance energy transfer (FRET). The energy of the excited state in FRET is transferred from the donor to the acceptor for subsequent emission via coulombic interaction [57]. This has the advantage of transferring energy over long distances. Therefore, the donor and the acceptor are not required to be directly attached. Indeed, they can be separated upwards of 100 Å by several unconjugated bonds in the EFC material. The key parameter governing FRET is the mutual overlap of the donor’s emission and the acceptor’s absorption spectra. The FRET efficiency, and hence the amount of emission from the acceptor, is contingent on the donor–acceptor spectral overlap integral. This can be leveraged for EFC use by either enhancing or reducing the donor–acceptor overlap by perturbing the energy states of either the donor or the acceptor electrochemically (Figure 4) [58,59,60].

In contrast to FRET, the Dexter exchange mechanism occurs over short distances (<10 Å). Typically, the emitter and the deactivator must be in proximity for deactivation to efficiently occur. The emitter transfers its excited electron to the LUMO of the deactivator with the simultaneous transfer of an electron from the HOMO of the deactivator to the emitter’s HOMO (Figure 2 right panel). While efficient excited-state deactivation via this pseudo-electron transfer mechanism requires the exergonic alignment of the energy levels, efficient transfer can also be achieved when the donor and acceptor are directly coupled. This is due to the large effective concentration (M) of the acceptor compared to the concentration of photoinduced excited states (μM) when the donor and acceptor are covalently coupled. 

The photoexcitation of an EFC device is carried out at a discrete wavelength, usually the maximum of the most red-shifted absorption of the EFC material. The absorption of the electrofluorochrome at a given excitation wavelength decreases concomitantly with its electrochemical depletion. This is because the absorption of the electrochemically generated intermediate generally does not overlap with the absorption of the neutral electrofluorochrome. It is possible to exploit the nonoverlapping and discrete absorption of the electrofluorochrome’s neutral state and its electrochemically generated intermediate to modulate its emission intensity by varying the excitation wavelength. In this case, the perceived emission decreases because there are fewer electrofluorochromes in the original state that are photoexcited owing to their conversion to the redox intermediate. The fluorescence is subsequently restored to the original resting state of the electrofluorochrome upon the electrochemical neutralization of the intermediate. This emission modulation process is not reliant on a change in the photoexcited deactivation process. Nonetheless, it is a reliable and efficient means of achieving high emission contrasts, provided that the absorption of the resting and electrochemically generated states of the electrofluorochrome are nonoverlapping. 

## 4. Molecular Electrofluorochromes

The performance requirements of electrofluorochromes used for the operation of EFC devices are as follows: (i) strong emission in the solid state, (ii) reversible redox behavior within the electrochemical limits of the device (iii), the quenching of the excited state by one of the photoinduced mechanisms outline above, (iv) emission intensity modulation with an applied potential, and (v) the excited state is not quenched by the electrolyte. Molecular electrofluorochromes are ideal for accurately establishing the effect of molecular structure on both the emission and redox properties of fluorophores. More importantly, they can be accurately evaluated to determine whether they meet the performance requirements for their ultimate use in functioning EFC devices. The knowledge acquired from evaluating molecular fluorophores can ultimately be used to rationally design electrofluorochromes that yield high-performance EFC devices. While intrinsic fluorophores meet the emissive criteria in solution for their use as EFC active materials, they must be modified so that they satisfy all of the materials’ performance requirements. Two general strategies have been adopted to develop fluorophores that can be used in EFC devices. One approach is the development of molecular dyads. These consist of an electroactive component that is covalently coupled with an intrinsic fluorophore. The other method relies on the intrinsic electroactivity of extensively conjugated fluorophores. The various structures developed and investigated in accordance with two design strategies of (A) covalently coupled electroactive fluorophores (molecular dyads) and (B) consolidated redox-active fluorophores are surveyed accordingly. Both methods are reliant on a photoexcited quenching mechanism that can be regulated electrochemically.

### 4.1. Covalently Coupled Electroactive Fluorophores

**Figure 5:**
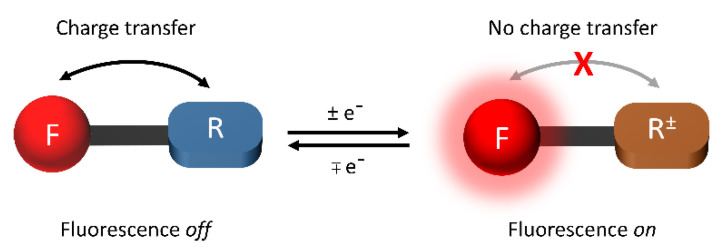
General structure of intrinsic fluorophore (F) covalently coupled with an electroactive component (R) of a molecular dyad and the intramolecular charge exchange mechanism that is responsible for electrochemically mediating fluorescence intensity modulation.

Conventional fluorophores are typically electrochemically inactive within the operating window of EFC devices. Therefore, the coupling of an electroactive moiety to a fluorophore is required to confer the requisite reversible redox activity to the emitter within the operating range of a functioning device. Toward this end, molecular electrofluorochromic dyads consist of an intrinsic emitter and a redox-active moiety that are covalently attached. The coupling of emitters and redox-active segments can be performed either with π-conjugated or -saturated alkyl linkers. Regardless of the type of coupling, the intrinsic emission of the emitter segment is reversibly modulated by electrochemically activating the redox segment that either sustains or suppresses the energy transfer or charge transfer mechanisms (schematically represented in Figure 5). 

**Figure 6:**
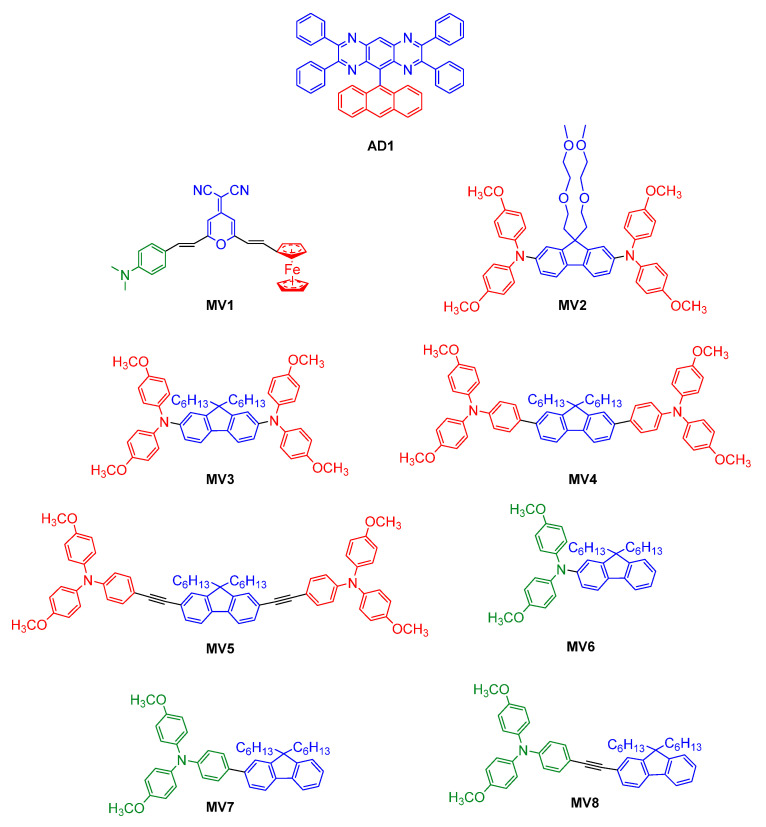
Molecular dyads and mixed-valence fluorophores consisting of a fluorophore (blue) and electroactive segments (red and green) examined as molecular electrofluorochromes [53,64,65,66].

Electroactive units that have been covalently attached to molecular fluorophores include triphenylamine [37], ferrocene [61], and tetrazine [62], among many others. Various conventional fluorophores have also been pursued with respect to their use as EFC materials. Success has been achieved with anthracene, carbazole, and dibenzofuran derivatives [63]. Anthracene has been used in electrofluorochromic materials owing to its electron-donating property. The tetraphenylpyrazino quinoxaline of **AD1** (Figure 6) prepared by S. Kim et al. was selected because of its reversible reduction and electrochemical robustness [64]. Anthracene was chosen as a donor (D) to couple with the quinoxaline acceptor (A). The covalently attached D–A pair was decoupled in the ground state because of its near orthogonal conformation (Figure 3). Photoexcitation coplanarized the two aromatics, resulting in their orbital coupling. This promoted an emissive TICT state. A large Stokes shift of 8700 cm^−1^ was partly indicative of coupling between the D and the A segments upon the photoexcitation of the tetraphenylpyrazino quinoxaline–anthracene dyad (**AD1**). Quinoxaline’s electrochemical reduction suppressed the otherwise emissive TICT state, and no fluorescence was observed. Indeed, the electrochemical generation of a radical anion led to fluorescence quenching. The fluorescence quenching was reversible over 12 cycles along with consistent maxima and minima of d(fluorescence)/dt. 

Ferrocene has also been widely used as an electroactive moiety in EFC devices because of its redox activity and chemical and electrochemical stability [67,68]. Its relatively low oxidation potential and its reversible oxidation are ideal for quenching the emission of fluorophores by PET with an applied potential [61]. This approach was adopted by J. Yang et al. who coupled an *N*,*N*’-dimethyl-aniline donor fluorophore with an electroactive ferrocene acceptor to afford a multivalent emitter (**MV1**, Figure 6) [65]. The fluorescence of **MV1** was quenched by PET from the donor to the acceptor when ferrocene was in its +2 oxidation state. The electrochemical oxidization of ferrocene to its +3 oxidation state suppressed PET and the intrinsic emission of the donor was restored. The emission could be reversibly quenched by electrochemically reducing the +3 oxidation state to the original +2 oxidation state.

Triphenylamine (TPA) is the organic counterpart of ferrocene. It has come to play a major role in electrofluorochromic materials, and it is the principal electroactive moiety for coupling with emitters. Its electron richness is also suitable for the promotion of intramolecular electron transfer. This process can shift the emission wavelength when triphenylamine is coupled with emitters. An advantage of using TPA is that its radical cation is non-fluorescent [69,70,71]. Therefore, the change in the intrinsic emission of the emitters with applied potential can be followed without the overlapping emission of the electrochemically generated intermediates. The intrinsic emission of the emitter can be readily modulated by covalently coupling the electroactive TPA. The latter was indeed the case with the end-terminated triphenylamine of the fluorescent fluorene **MV2** prepared by G. Corrente et al. (Table 1) [66]. The photoinduced emission of **MV2** could be reversibly deactivated and activated by electrochemically oxidizing the terminal triarylamine and reducing the oxidized state, respectively. A working EFC device was fabricated with **MV2** as the active layer and the device displayed high electrochemical stability and a consistent fluorescence contrast ratio upwards of 4500 cycles. The Φ_fl_ of the device was also greater than that in solution. The performance enhancement of **MV2** was from its restricted intramolecular motion excited-state deactivation that was suppressed in the viscous electrolytic gel in the operating device. The robustness of the material with respect to its ability to maintain performance in the operating device was demonstrated by a decrease in the emission contrast ratio of only 50% after 18,000 electrochemical oxidation/neutralization cycles [66]. The stability of the triphenylamine–fluorene during the extended redox switching cycles of the applied potential is exceptional compared to other systems.

Using the same triphenylamine–fluorene framework, A. Capodilupo et al. investigated the dependence of the intervalence charge-transfer (IVCT) transitions and the electronic coupling between the redox centers (π–π*) on the π-conjugated bridge length [53]. Their study focused on donor-π-donor (D-π-D) (**MV3–5**) and single donor (D-π) (**MV6–8**) electroactive fluorophores. These fluorophores were prepared to evaluate their solvent-dependent absorption and emission properties. The **MV** emitters were also prepared to provide information about the electron transfer energetics and the different types of transfer, such as π–π* and charge transfer (CT). **MV3–5** had an intense IVCT in the NIR region, which was exploited to quantify the nature of the electron-coupling process that occurred. The **MV3** derivatives also had ideal EFC properties, including high Φ_fl_ (>90%), high IVCT molar absorptivity coefficients (>40,000 M^−1^ cm^−1^), and their optoelectronic properties could be tuned by varying the length of the π-conjugated bridge. **MV3–5** emitted at 422, 464, and 424 nm, with Φ_fl_ = 70, 92, and 90%, respectively. The single donors (**MV6–8**) emitted at 441, 472, and 472 nm, with Φ_fl_ = 67, 89, and 97%, respectively. 

**Figure 7:**
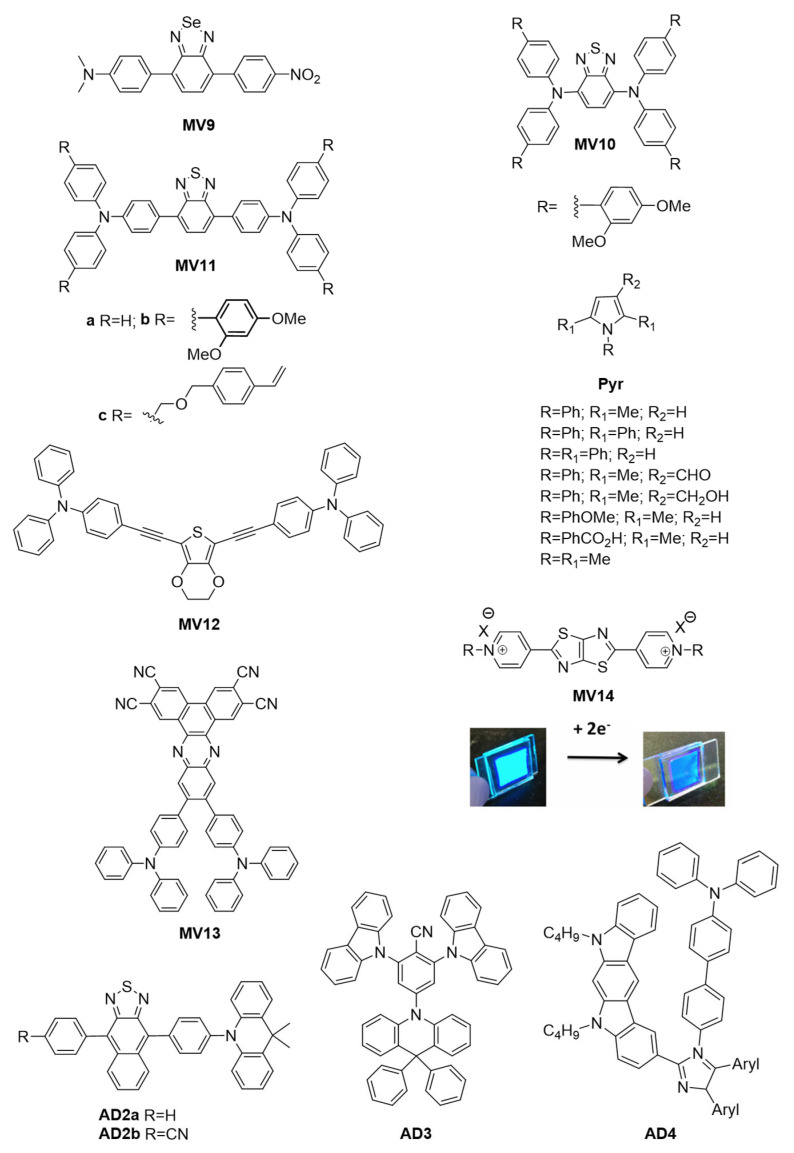
Mixed-valence **MV9–MV14** and **AD2a,b–AD4** investigated as electrofluorochromic materials [54,55,72,74,76,79,80,81,82]. Inset image of **MV14** EFC device in the photoexcited on (**left**) and off (**right**) states [83]. Reproduced with permission from ref. [83]. Copyright 2017 American Chemical Society.

Benzoselenadiazole (**MV9**, Figure 7) was also rationally designed to undergo changes in both emission wavelength and emission intensity with solvent polarity [72]. Intramolecular charge transfer between the electronic terminal groups was favored in polar solvents. This was based on the well-known strong emission and solvatochromism of its benzothiadiazole counterpart, **MV11a** [73]. Indeed, its intrinsic emission was exploited to develop a styrenic monomer counterpart, **MV11c,** that could be thermally polymerized directly on the device’s electrode [74]. The emission that was solvent-contingent, with Φ_fl_ upwards of 85%, could be carried over to develop a thin film that was visibly fluorescent. The intrinsic fluorescence of the electroactive film of **MV11c** was electroactive owing to the presence of triphenylamines, resulting in fluorescence modulation with an applied potential. Unsymmetric derivatives of **MV11** consisting of an electroactive triphenylamine and an emissive pyrene with either a benzooxadiazole or benzothiadiazole core were also electrofluorochromic, with a solid-state emission of 7% [75]. These devices could be switched upwards of 100 times with 85% emission recovery. This was extended towards the dimethoxy benzothiadiazole counterparts **MV10** and **MV11b**. These highly emissive benzothiadiazoles developed by G. Corrente et al. currently hold the electrofluorochromic contrast ratio record of 1230 [55]. Via chemical oxidation with SbCl_5_, it was also demonstrated that a persistent green emission of the electroactive benzothiadiazoles stemmed from a radical cation. This unprecedented room-temperature emission confirmed that the radical cation did not quench the fluorescence of the intrinsic emitter; rather, it contributed to the perceived emission albeit it weakly. Toward this end, L. Zheng et al. demonstrated that simple *N*-substituted pyrroles (**Pyr**) could readily be oxidized in air [76]. The resulting radical cation was also emissive, with its emission occurring in the visible spectrum. Both the emission wavelength and Φ_fl_ were somewhat contingent on the substitution of the pyrrole with Φ_fl_ ≈ 5–11%. Similar to G. Corrente et al., the identity of the pyrrole radical cation was confirmed by chemical oxidation albeit with silver hexafluoroantimonate. The identity of the radical cation was further confirmed by EPR measurements. Although electrofluorochromism was not studied by L. Zheng et al., it would be possible to exploit the pyrrole’s electrochemical activity to anodically generate an emissive radical cation for an electrofluorochromic response in both a solution and in the solid state. This approach has the advantage of not requiring a conjugated intrinsic fluorophore since the desired visible emission occurs because of the narrow SOMO–LUMO energy gap of the radical cation. Therefore, simpler molecular structures can be used, which provide the benefit of not requiring multistep synthesis. The fluorescence that is activated when **Pyr** is oxidized to its radical cation contrasts with that of radical imaging probes [77,78]. These sensors rely on PET between the fluorophore and the radical cation to quench emission. The intrinsic emission of the fluorophore can be restored upon the electrochemical depletion of the radical cation [78].

Similar to **MV11**, **MV9** (615 nm) had the most intense emission in hexane with Φ_fl_ = 71%. The fluorescence of **MV9** could also be reversibly deactivated with an applied potential of ca. +420 mV vs. the reversible ferrocene/ferrocenium (Fc/Fc^+^) couple used as a reference. Similarly, its visible color could be reversibly switched from its yellow/orange resting color to its pale-violet oxidized state with a potential of 1.1 V vs. Fc/Fc^+^. The emission wavelength and intensity of **MV9** could be adjusted by varying the polarity of the solvent. The emission intensity decreased two-fold in diethyl ether compared to hexane and the emission wavelength shifted to 685 nm from 615 nm. Meanwhile, the emission was nearly quenched in acetone, with the emission shifting to the near infrared (810 nm) [72]. M. Wałęsa-Chorab et al. developed **MV12** to examine the effects of the electronic-rich central aromatic (thiophene vs. ethylenedioxythiophene (EDOT)) and the substitution of the terminal TPAs on the EFC properties [84]. Unlike the emitters synthesized by A. Capodilupo et al. that exploited the intrinsically fluorescent fluorene core [53], the fluorescence of **MV12** was from the conjugated alkyne framework with an electron-rich central heterocycle. Using **MV12** as the active component in the EFC device, the fluorescence could be reversibly quenched upwards of 96% during 30 s of applied potential. The original fluorescence slowly began to decay after 30 min of switching the potential. Conversely, the 30% color contrast of the device was maintained, and it only began to decay after 1780 cycles of switching the potential. 

U. Balijapalli et al. also used the donor–acceptor approach to prepare an electron-deficient dibenzo[a,c]phenazine-2,3,6,7-tetracarbonitrile core that was capped with two TPA donors (**MV13**) [79]. Owing to its intrinsic emission, **MV13** was used as the emitter in OLEDs, with the device continuously emitting upwards of 168 h at 10 mA/cm^2^. Devices fabricated with **MV13** had long lifetimes (>600 h) with a moderate Φ_fl_ (40%). The color and emission of **MV13** could be adjusted through doping. The color of **MV13** could be switched from its intrinsic black to red by doping it with 1 wt% of 4,8-bis [4-(*N*,*N*-diphenylamino)phenyl]benzo [1,2-c:4,5-c’]bis [1,2,5]thiadiazole. The color change could be extended to orange with additional doping [79]. In a similar manner, Y. Gao et al. synthesized two deep-red emitters: **AD2a** and **AD2b**. While the emitters had a low Φ_fl_, namely, 4% (**AD2a**) and 9% (**AD2b**), appending a nitrile group to the conjugated system improved the Φ_fl_ [80]. The obtained increased emission was produced by an aggregation-induced emission effect (vide infra) resulting from hydrogen bonding between two separate molecules of **AD2b**. Their emission could also be varied with doping. Indeed, the emission of **AD2a** and **AD2b** shifted to 588 and 612 nm via doping, along with increasing their Φ_fl_, to 47% and 28%, respectively. Z. Cheng et al. developed a similar donor–acceptor **AD3** whose acceptor moieties were shielded from the carbazole [81]. The compounds were designed to have a pure-blue emission and not undergo ICT that would otherwise interfere with pristine emission. The intrinsic emission of a carbazole derivative, indolocarbazole (**AD4**), was further exploited by Y. Liu et al. [54]. Their emitter was coupled with an electroactive TPA via various imidazole cores, with the unconjugated frameworks undergoing reversible photoemission quenching over 300 cycles.

Dipyridinium thiazolo [5,4-d]thiazole has emerged as an alternative intrinsic fluorophore because it emits appreciably [83,85]. It is also electroactive due to its terminal pyridines. T. Adams et al. capitalized on these properties along with the fluorophore’s straightforward synthesis to prepare a series of pyridine-substituted electrofluorochromic materials (**MV14**) [82]. These had the benefit of being fluorescent in their solid state. The emission contrast ratio between the on (0 V) and off (2.5 V) states of their operating devices was upwards of 143-fold and contingent on the substitution of pyridine. This pyridine substitution also affected the on/off emission ratio over the course of reversibly switching the applied potential. A consistent fluorescence on/off ratio was observed over 25 cycles of switching the applied potential, wherein R = benzyl, with chloride counterions, whereas the ratio increased when R = trimethylaminopropyl.

### 4.2. Consolidated Redox-Active Fluorophores

The extended conjugated framework of fluorophores that emit in the visible and NIR regions typically have the beneficial property of being electroactive. Such emitters are not reliant on a covalently linked electroactive moiety for introducing redox activity within the electrochemical operating window unlike their molecular electrofluorochromic dyad counterparts. The benefit of consolidating the redox activity within the conjugated framework of the fluorophore is the reduced number of steps that are required for their synthesis. The redox potential of the fluorophore can be varied by incorporating electronic groups into the conjugated framework. The electron richness of the fluorophores provides the means to further adjust the redox potential. For example, the cathodic channel can be accessed by using electron-deficient heterocycles such as pyridine, tetrazine, and benzothiadiazole. In contrast, an anodic behavior can be activated by using electron-rich heterocycles such as thiophene and EDOT. The development of fluorophores containing both electron-deficient and -rich heterocycles facilitates the tuning of the emission wavelength and the modulation of the emission intensity by both electrochemical oxidation and reduction without relying on the covalent coupling of redox-active moieties. The strides made towards electroactive fluorophores that meet the requirements for their use in electrofluorochromic devices are outlined in the following subsections.

#### 4.2.1. Redox-Active Organic Fluorophores

**Figure 8:**
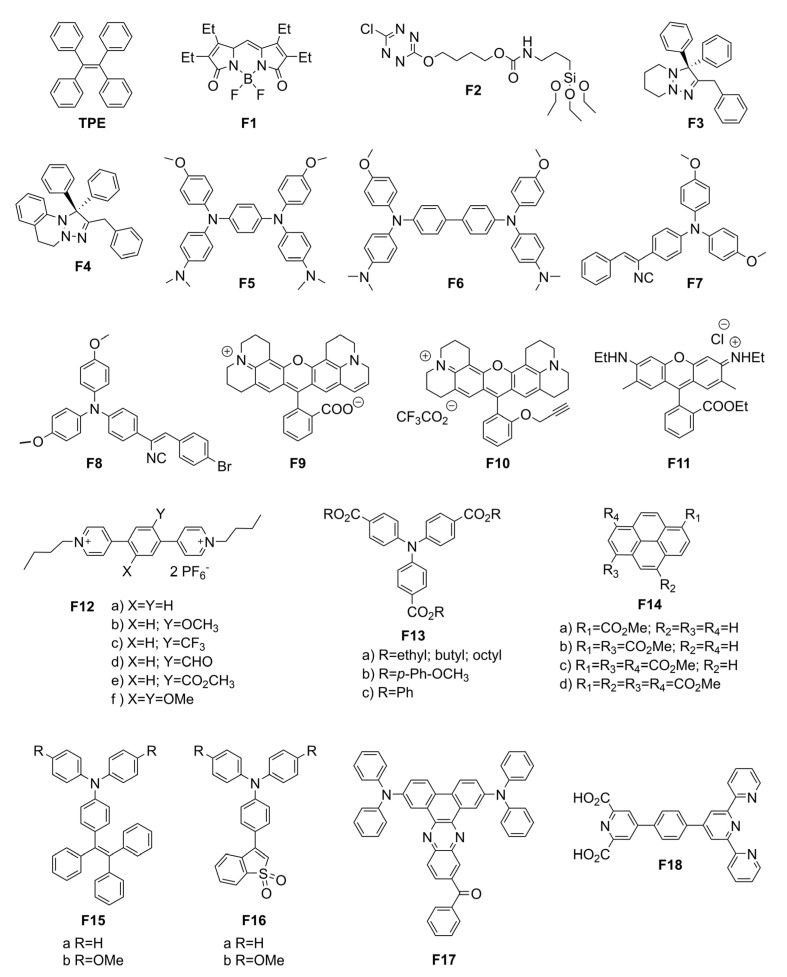
Representative examples of consolidated intrinsically electroactive organic fluorophores used as EFC materials [87,88,89,90,91,92,93,94,95,96,97,98,99].

Boron dipyrrin (BODIPY) is widely used in bioimaging applications due to its intrinsic fluorescence [86]. Its emission is in part generated via *N*-heterocyclic coordination with the Lewis acid boron. This interaction planarizes the fluorophore and extends the conjugation of the framework [86]. R. Gautam et al. exploited the emission of BODIPY to develop **F1**, whose emission intensity could be modulated electrochemically (Figure 8) [87]. The radical anion, [(pdp)BF_2_]^•–^, which was formed by an electrochemical single-electron transfer, quenched the intrinsic emission of **F1**. Fluorescence could be restored by regenerating the neutral state through the electrochemical oxidation of the radical anion.

Tetrazines are similarly interesting nitrogen-containing aromatics with respect to electrofluorochromism owing to their intrinsic fluorescence and redox activity. An advantage offered by these heterocyclic aromatics is their electron deficiency, which allows them to be electrochemically reduced. This was exploited by Guerret-Legras et al. who developed monolayers of the tetrazine **F2** (Figure 8) [88]. This was achieved by covalently bonding a coating of the fluorophore to ITO-coated glass via a triethoxy siloxane linker. Their triazolines were electrofluorochromic, with the fluorescence activity at 560 nm being reversibly suppressed, and with fluorescence turn-off/turn-on potentials of −0.65 and 0 V vs. Ag/AgCl, respectively. Other nitrogen-containing aromatics that are ideal electrofluorochromes because of their inherent electroactivity and moderate fluorescence are triazolines. A. Suleymanov et al. examined several polysubstituted triazoline derivatives with respect to their electrofluorochromism [89]. While their triazolines did not fluoresce in solution, the solid-state Φ_fl_ values of **F3** and **F4** were ca. 6% and 3%, respectively. Nevertheless, the emission of these triazolines could be observed despite their relatively low absolute Φ_fl_. Indeed, **F3** and **F4** were electrofluorochromic, and their emission could be reversibly quenched by applying an off/on potential of 200 and 160 mV vs. Fc/Fc^+^, respectively. 

**Figure 9:**
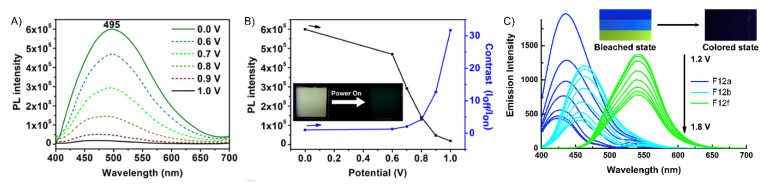
Electrofluorochromism of **F6** in solution (**A**) and in a full device (**B**). Electrofluorochromism of **F12a**, **b**, and **f** in a full-device configuration (**C**) [93,98]. (**A**,**B**) Reproduced with permission from ref. [98]. Copyright 2019 American Chemical Society. (**C**) Reproduced with permission from ref. [93]. Copyright 2020 Elsevier.

Other nitrogen-containing derivatives that continue to play important roles in electrochromics, and hence electrofluorochromics, are triphenylamines (TPA). Their widespread use in such forms of electrochemically mediated property switching is due to their relatively low oxidation potential and reversible oxidation [100]. TPAs further undergo a visible color change that is typically blue with an applied potential, with the color of the neutral state being contingent on their substitution [101]. Another reason for TPAs’ widespread use is that they can be readily modified by being coupled with various functional groups and aromatics. Their color, emission, and redox properties can be adjusted by their structural modification. Such modification further makes TPA a dual-state emitter, i.e., fluorescing in both solution and the solid state [102,103]. The latter is of the upmost importance for their use in solid-state operating devices. Towards this end, J.-T. Wu et al. studied TPA derivatives **F5** and **F6** as fluorochromes in working devices with heptyl viologen as the ion storage layer [98]. The devices fabricated from **F5** and **F6** (Figure 9A,B) had high fluorescence contrast ratios of 31 and 21, respectively, and excellent stability, and **F6** had moderate fluorescence off/on switching times of 9.7 s and 4.9 s.

S.-Y. Chen et al. reported four electroactive cyanostilbenes and TPA-containing derivatives with different substituents [90]. Both **F7** and **F8** displayed EFC properties in operating devices with heptyl viologen as an ion storage layer. The fluorescence off/on response times of the EFC device with **F7** were 21 s and 11 s, respectively, while similar off/on behaviors were observed for **F8**, namely, 14 s and 7 s, respectively. The contrast ratio of fluorescence for **F7** and **F8** reached 6.75. A spectroscopic advantage of **F7** and **F8** is their aggregation-induced emission (AIE), which facilitates their increased emission when cast as thin films. AIE-active molecules are typically aromatics that have large degrees of freedom. These modes readily promote excited-state energy dissipation by nonradiative means such as bond rotation and vibration. Thus, the emission is quenched when the AIE-active compounds are diluted in solution. In contrast, when the aggregation-induced emitters are either aggregated in solution or in the solid state, the otherwise efficient emission deactivation pathways are suppressed. The fluorescence yield increases because the restricted intramolecular motion deactivation pathways of the excited state are no longer active. Tetraphenylethene (**TPE**, Figure 8) is the most widely used AIE fluorophore [104,105,106]. It fluoresces minimally in solution, but its emission is significantly increased when aggregated in solution. It also emits when it is either in the form of a powder or cast in thin films without a filler. Its prevalent use as a universal solid-state emitter is not only a result of its intrinsic intense emission when not diluted in a solvent but also because it can be readily substituted. Therefore, it can be covalently attached to a wide range of materials to render them fluorescent in the solid state, thus satisfying a key criterion for their use as EFC materials in operating devices. 

Rhodamines are intrinsically fluorescent nitrogen derivatives that are also suitable for electrofluorochromic applications. M. Čížková et al. assessed the suitability of rhodamine derivatives **F9** and **F10** for solution-based EFC [91]. While the rhodamines had good switching times, they suffered from limited electrofluorochromic reversibility. Slanina and Oberschmid further evaluated rhodamine **F11** with respect to its capacity to reversibly switch between its fluorescence on and off states [92]. The rhodamines were qualitatively found to maintain their EFC behavior. The intrinsic red emission of a rhodamine derivative was further capitalized by W. Zhang et al. to develop a simultaneously emitting red, blue, and green pixelated electrofluorochromic device [107]. In this case, the blue and green emissions of the RGB device were provided by triphenylamine and fluorescein, respectively. Reversible quenching of the red and green emissions was achieved by electrochemically induced proton-coupled electron transfer.

Viologens are another group of interesting nitrogen-containing electroactive fluorochromes. This was demonstrated by Y. Shi et al. who synthesized six viologen derivatives, **F12a–f** [93]. The excited-state quenching of the cations could be suppressed (vide infra) by separating the charged pyridines with different disubstituted phenyls. As a result, **F12a–f** were fluorescent (Figure 9C). Devices prepared using these viologens displayed remarkably fast response times and had consistent electrochemical properties. M. Wang et al. developed other nitrogen-containing, deep-blue electrofluorochromic materials composed of TPA and a triazole-dibenzonitrile [108]. Through their HOMO/LUMO DFT calculations and by studying the dopant’s effect on the materials’ fluorescence, they could modulate the fluorescence lifetime from 146 ns to 5.08 ns via doping. The emission wavelength could also be adjusted by doping, with the emission of the films red-shifting upwards of 85 nm when doped, corresponding to a deep-blue color (≈470 nm).

W.-j Zhang et al. further exploited the intrinsic fluorescence of TPA and modulated its reduction potential and emission wavelength by substituting TPA with various 4,4′,4″-benzoates (**F13a–b**) [94]. The emission wavelength at ca. 425 nm could be varied ± 10 nm and the Φ_fl_ could be tuned from 2 to 32% depending on the ester. Esters were also used by F. Li et al. to confer a reversible electrochemical reduction to an intrinsic pyrene emitter (**F14**) [99]. The cathodic potential could be modulated depending on the number of ester substitutions. The emission of **F14a** and **c** could be quenched by applying a negative potential for 200 and 120 s, respectively. The electrofluorochromic behavior of **F14c** was fully reversible. This contrasted with **F14b**, whose emission could be quenched electrochemically. Although the pyrene esters were electrofluorochromic, this desired behavior was eclipsed by their electrochromic performance. The pyrenes had sub-second coloration and bleaching times, and the ester could maintain upwards of 90% color retention and 65% color contrast after 2100 switching cycles.

The solid-state emission of TPA can also be modulated by both electronic effects and its covalent attachment to AIE emitters. The emission of TPA can be tuned to span 100 nm depending on the substitution employed. This was demonstrated by the TPA derivatives **F15a–b** and **F16a–b** developed by H. Lin et al., whose solid-state emission varied between 462 and 560 nm and whose Φ_fl_ ranged from 25 to 98% [95]. The fluorescence contrast ratio of the full devices prepared from **F15–F16** was consistently ca. 7. The TPA fluorophores could sustain multiple redox cycles with minimal degradation of their intrinsic emission as per their 98% photoemission recovery after 100 cycles of switching the applied potential. 

B. Sk et al. prepared a central dibenzophenazine with two terminal TPAs (**F17**) [96]. ICT from the flanking TPAs to the electron-deficient dibenzophenazine within the conjugated donor–acceptor-donor arrangement led to a visible red emission. The Φ_fl_ was solvent-dependent, ranging from 40% in apolar solvents to 7% in aprotic polar solvents. The radical cation, formed by two one-electron oxidations of the TPAs, suppressed the ICT. This resulted in the quenching of the fluorescence. This was confirmed both by chemical and electrochemical oxidation. The fluorescence could be reversibly quenched, taking about 85 s to achieve a maximum quenching. The chemically generated dication also completely quenched the fluorescence. 

The terpyridine derivative (**F18**) was designed to act as a gelator by S. Halder et al. [97]. It formed a hydrogel at moderate concentrations, and it was conductive following the addition of NaCl. The intrinsic fluorescence of **F18** could be quenched in an operating EFC device with an applied potential of −3.5 V. The emission recovery in the device was slow, and it took upwards of 2 min to regain 80% of the original emission. Nonetheless, the activation/deactivation of emission was reversible during at least five cycles of switching the applied potential. 

It is evident that nitrogen-containing aromatics are suitable as consolidated electrofluorochromes owing to both their intrinsic fluorescence and electroactivity. Their redox property, and most importantly, their emission wavelength can be tailored by adjusting their molecular structure. Amines are an equally important functional group with which to induce electroactivity in materials that would otherwise be insulators, while also providing the means to tailor a material’s optical and emission properties. Owing to its electron donor property, TPA can also promote photoinduced ICT with acceptors. This opens the pathway for controlling ICT fluorescence electrochemically by oxidizing the electron-rich amine. TPA and nitrogen heteroaromatics are viable choices to include in the rationale design of molecular electrofluorochromes.

#### 4.2.2. POMS Redox-Active Fluorophores

**Figure 10:**
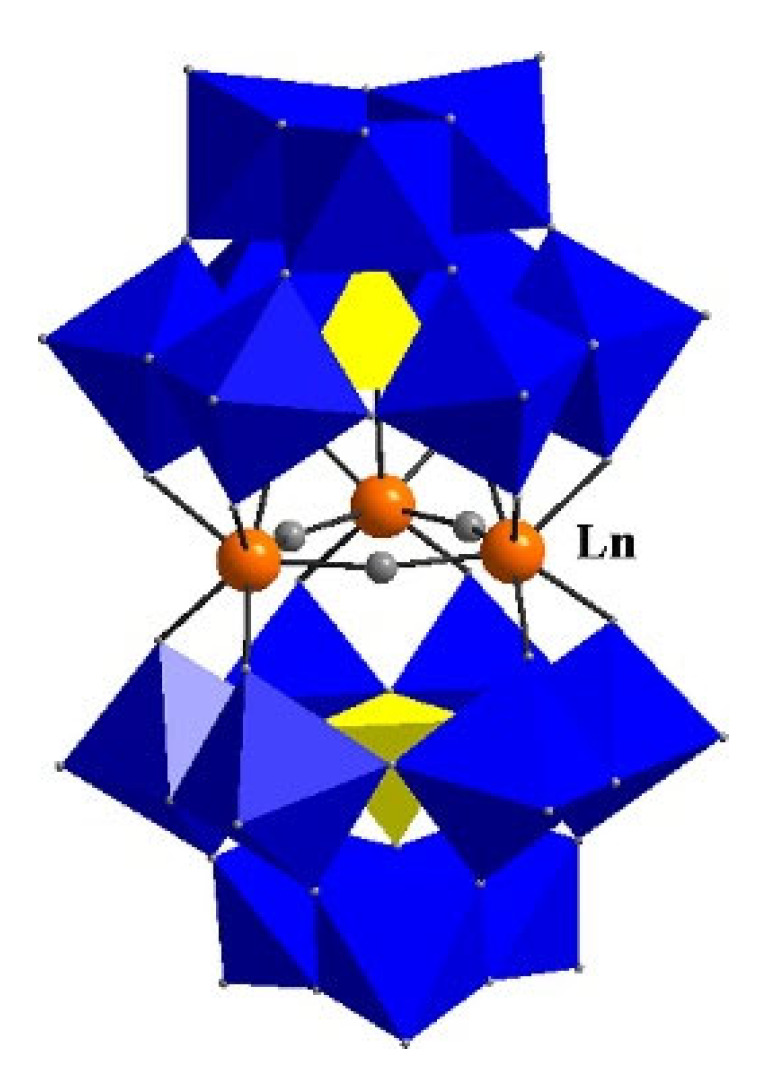
Structure of a Ln_3_O_3_(SiW_9_O_34_)_2_^17−/15−^ POM with the WO_6_ octahedra shown in blue and the SiO_4_ tetrahedra shown in yellow [58]. Reproduced with permission from ref. [58]. Copyright 2020 Elsevier.

The intrinsic redox activity of metals has been exploited for the electrofluorochromism of metal complexes [40,44,109]. Such has been the case with ruthenium [110,111,112,113], europium [114,115], and iridium [116,117] complexes, along with metal–organic frameworks [118] that are electrofluorochromic. Other metal-encompassing materials that are emerging for use as electrofluorochromes are polyoxometalates (POMs) [58]. These are clusters of transition metals with atoms that are spatially organized in different arrangements. A representative POM is shown in Figure 10. Their sandwich structures are highly soluble, and they have good thermal resistance. Owing to their constitutional metals, POMS are both electroactive and they have high electrochemical stability. However, their inherent crystallinity limits the use of POMS in devices when in their neat form. To overcome this shortcoming, POMS are usually combined with an organic matrix such as polymers. Such composites are easily fabricated and they are chemically and mechanically robust. For example, a composite film was prepared by W. Gao et al. by mixing a samarium tungstophosphate K_3_Cs_8_ [Sm(PW_11_O_39_)_2_]·10 H_2_O(SmPW_11_) POM with poly(diallyl dimethyl ammonium chloride; PDDA) and poly(ethyleneimine; PEI) [119]. Both PDDA and PEI played the role of a polymer matrix in which the otherwise crystalline POM was dispersed, resulting in a film of [PDDA/P_2_W_18_]_10_/[PDDA/SmPW11]_60_ and [PEI/P_2_W_18_]_10_/[PEI/SmPW_11_]_60_(PEI film). Multilayers of POMS/polymer matrix composite films suitable for devices could also be prepared from either ErSiW_9_ or SmSiW_9_ acting as the POM and C_30_H_31_N_6_^+^ (JGB) as the matrix. The [ErSiW_9_/JGB]_n_ and [SmSiW_9_/JGB]_n_ composites were fluorescent. W. Gao et al. also developed a GO@HOPTS matrix from 8-hydroxypyrene-1,3,6-trisulfonic acid trisodium salt (HOPTS) and graphene oxide (GO) [120]. Blending the matrix with the tungsten POM led to robust {[(PDDA/P_5_W_30_)_5_/[(PDDA/P_5_W_30_)_5_/PDDA/GO@HOPTS]_15_/(PDDA/P_5_W_30_)_5_]} composites. While POM composites are promising for electroluminochromic applications, the fine-tuning of their structural composition and their compounding are still required to improve their properties before they can rival the properties of their organic counterparts. However, this is achievable because of the structural diversity that is possible with POMs. 

### 4.3. Understanding Design Principles of Molecular Electrofluorochromes

From the consolidated spectroscopic and redox data presented in Table 1, it is evident that molecular fluorophores can be converted into ideal electrofluorochromes. The integration of TPA provides the means to activate otherwise electrochemically inert fluorophores within a given electrochemical window while preserving the emitter’s intrinsic high Φ_fl_ (**MV** series). The emission wavelength of the fluorophore can be modulated upwards of 320 nm by incorporating TPA, as the redox-active component, into the conjugated framework of the fluorophore without affecting the desired reversible redox behavior of TPA. Indeed, reversible redox behavior consistently occurs at relatively low oxidation potentials (<1 V vs. SCE) that are ideal for mitigating the undesired redox-mediated degradation of the electrofluorochromes. Functioning electrofluorochromic materials with high performances, especially appreciable solid-state Φ_fl_, can be further designed by including functionalities that promote dual-state fluorescence, namely, emission in both solutions and in the solid-state. While TPE has become a workhorse for promoting solid-state emission, synthons that promote supramolecular solid-state contacts can also be used to promote emission in EFC films (**F6–8** and **F16**), along with structural modifications that promote restricted intramolecular motion (**MV2**) in solid-state devices. The emission range of the electrofluorochromic materials can be adjusted via structural modification while exploiting the design principles for solid-state emission. The replacement of TPA with either electron-deficient heterocycles (**AD1**, **F2**, **F9–11**, and **F18**) or electron acceptors (**F14**) renders electrofluorochromic materials cathodically active, thus allowing for the photoemission intensity to be modulated by applying a negative potential. Electrofluorochromic materials that can be both electrochemically oxidized and reduced are possible by combining TPA with electron acceptors (**F13**). Viologens are suitable aromatics for conferring reversible cathodic activity to the electrofluorochromic materials. These electron-deficient heterocycles can be rendered fluorescent by separating the charged bipyridines with various aromatics (**F12**), resulting in high-performance electrofluorochromic materials (**MV14**). Moreover, emission colors covering a large breadth of the visible spectrum are possible with π-donor–acceptor-conjugated fluorophores. Meanwhile, incorporating the electron-deficient benzothiadiazole into the fluorophore framework of electron-rich aromatics offers the advantage of carrying the intrinsic emission over to the solid state, which is a key performance target for functioning EFC full devices.

## 5. Electrofluorochromic Polymers

By combining intrinsic fluorophores with electroactive groups, molecular electrofluorochromic materials can be designed to have a high electrofluorochromic performance to enable functioning devices. The advent of readily accessible and diverse synthetic methods such as direct arylation polymerization has resulted in molecular electrofluorochromic materials that cover a wide range of aspects related to structural diversity, and in turn, a range of emission and redox properties [121]. Although the properties of molecular fluorophores can be tailored, their properties tend to degrade during prolonged device operation. Conversely, the performance of devices prepared from polymers is consistent during extended periods of device operation. As a result, there has been a surge in the use of polymers as EFC materials during the past five years. This is in part because of their electrochromic properties, which have been extensively investigated. Moreover, the design elements required to render polymers electrochromic, especially π-conjugated organic polymers, are well established and have been successfully used in electrochromic devices over the past 20 years [122,123,124,125,126,127,128].

The design principles for electrofluorochromic polymers are similar to their molecular counterparts. They must contain an intrinsic emitter and an electroactive component. The electroactive component must also be capable of regulating one of the photo-excited-state-quenching mechanisms. Ideal polymers for use in working EFC devices can be derived from molecular EFC components that are incorporated into the polymer either as pendant groups or directly as constitutional components of the polymer. Such EFC polymers are often unconjugated. These polymers exploit the EFC properties of the constitutional components. Meanwhile, the polymer component provides a film-forming capacity while also imparting both mechanical and electrochemical robustness. The electroactive and emissive monomers, consisting of complementary difunctional groups, can be coupled to form polymers via step-growth polymerization. Imides, amides, and ureas are among the most widely used functional groups with which to couple the electroactive and emissive monomers to synthesize polymers. This is in part due to their straightforward preparation via the thermal condensation of stoichiometric amounts of the corresponding difunctional monomers. A benefit offered by such unconjugated polymers is their limited intramolecular charge transfer. While charge transfer promotes molecular stability and orbital overlap, it quenches the fluorescence. Therefore, unconjugated polymers are potentially more intrinsically fluorescent than their conjugated counterparts (vide infra); providing a fluorophore is part of their constitutional makeup.

Conjugated polymers can similarly be prepared by the step-growth polymerization of difunctionalized complementary monomers via direct arylation polymerization [121,129,130,131,132], Suzuki coupling [133], and Kumada methods [134]. Although they can be used as electrochromes, they may not be ideally suited for use as electrofluorochromic materials. This is in part because of their narrow energy gap between the HOMO and LUMO energy levels that can result in efficient excited-state deactivation by non-emissive means. Their desired emission can also be self-quenched when cast as thin films. Therefore, increasing the layer thickness of electrochromic polymers exacerbates their fluorescence self-deactivation efficiency.

### 5.1. TPA-Containing Electrofluorochromic Unconjugated Polymers

Unconjugated EFC polymers can be grouped into two distinct categories: those containing TPAs to ensure redox activity and those reliant on electroactive moieties other than TPA. Like its molecular counterparts, TPA dominates the field of unconjugated EFC polymers, and it is the most abundantly used electroactive component. This is because it retains its desired reversible oxidation and its capacity to sustain multiple cycles of redox switching when incorporated into a polymer. It can also be derivatized with a range of functional groups for its subsequent polymerization. TPE has also become a key component that is integrated into EFC polymers to confer the requisite fluorescent behavior to the polymers. This is in part because it can be readily incorporated into polymers as either a pendant group or a constitutional component of the polymer owing to its straightforward modification. More importantly, polymer films containing TPE are highly fluorescent and, therefore, meet the fluorescent performance requirement to enable solid-state EFC devices. The polymers presented hereafter exploit the AIE effect of TPE that renders them suitable for use in electrofluorochromic devices.

**Figure 11:**
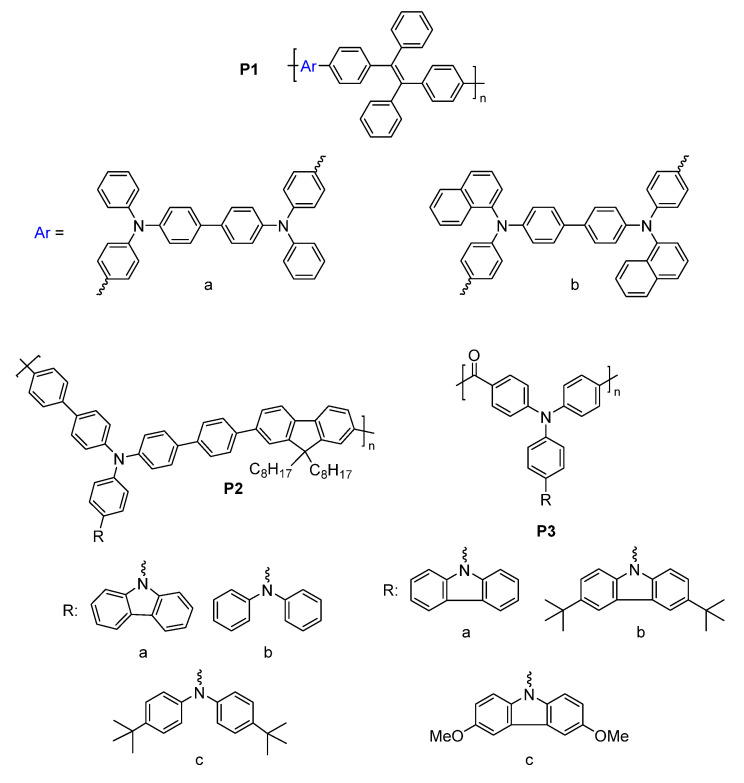
Representative structures of TPA-containing polymers used as electrofluorochromic materials [135,136,137].

**Figure 12:**
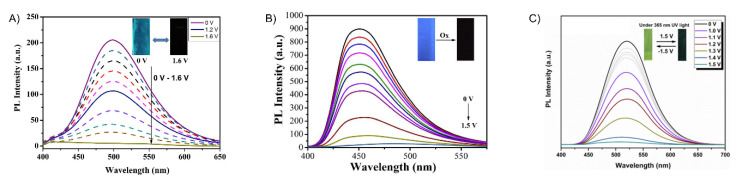
Electrofluorochromism of **P1b** (**A**) and **P2a** (**B**), and **P3b** (**C**) in a half-device configuration. Insets: photographs showing the fluorescence off/on with applied potential [135,136,137]. (**A**) Reproduced with permission from ref. [135]. Copyright 2019 Elsevier. (**B**) Reproduced with permission from ref. [136]. Copyright 2019 Elsevier. (**C**) Reproduced with permission from ref. [137]. Copyright 2019 Elsevier.

Q. Lu et al. designed four conjugated polymers that incorporated TPA as a constitutional component [135]. To overcome the fluorescence quenching caused by the narrowing of the energy-gap, they exploited the AIE effect of TPE. Their **P1a** and **P1b** polymers (Figure 11) were highly fluorescent, with Φ_Fl_ values of 69 and 75%, respectively. When used in EFC devices, the fluorescence-quenching and restoration times of the polymers were 1.6 s/2.6 s (**P1a**) and 4.2 s/3.6 s (**P1b**) for the colored and bleached states, respectively. The intense emission of the polymers was due to the AIE effect, especially with respect to the TPE derivatives (Figure 12A). **P1b** had the highest fluorescence contrast ratio of 41 and a color contrast ratio of 16% over 50 switching cycles at 20 s intervals [135]. C. Yang et al. further designed and prepared three copolymers derived from fluorene and TPA: **P2a**, **P2b**, and **P2c**. In their case, the TPA employed was a constitutional component of the polyamide, and the emitter was a pendant fluorene [136]. The devices prepared from **P2a** were electrofluorochromic between 0 and 1.5 V (Figure 12B). A similar polyamide with TPA as its constitutional component and incorporating pendant emitters was designed and prepared by Y. Han et al. [137]. They examined the effect of the substitution of the pendant carbazole emitters on the synthesized material’s properties. The films of their **P3a**, **P3b** (Figure 12C), and **P3c** were emissive and electrofluorochromic [137]. **P3b** had a fast fluorescence response time to quench the emission of 1.42 s. While **P3b** showed a high transmission ratio of 87%, the emission intensity of the deactivated state of the film decreased after only a dozen switching cycles, demonstrating its poor resistance towards repetitive cycling of the applied potential. 

#### 5.1.1. Polysilsesquioxanes and Polyimides 

**Figure 13:**
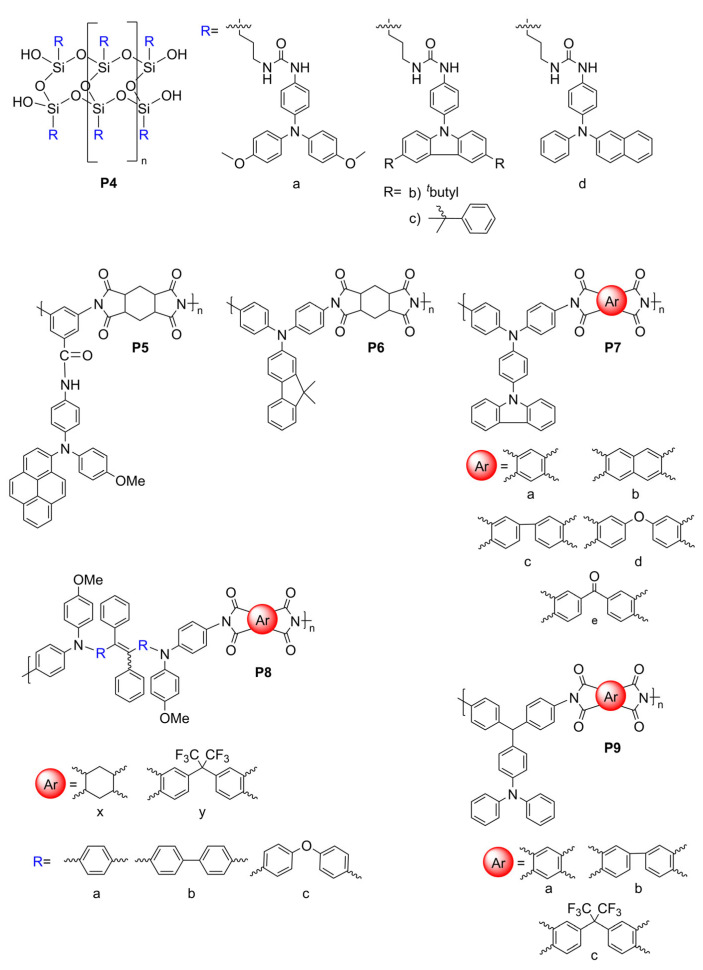
Polyimide derivatives used as electrofluorochromic materials [138,139,140,141,142,143].

**Figure 14:**
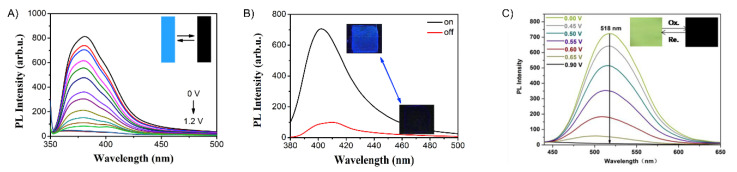
Electrofluorochromism of **P4b** in a half-device (**A**), **P4a** in a full device (**B**), and **P5** in half-device (**C**) [138,139]. Insets: photographs showing the emission on/off with applied potential. (**A**,**B**) Reproduced with permission from ref. [138]. Copyright 2020 Elsevier. (**B**) Reproduced with permission from ref. [139]. Copyright 2019 Elsevier.

W. Zhang et al. synthesized four electrofluorochromic polysilsesquioxanes [138]. This was performed by exploiting the reactivity of isocyanates to covalently attach amine-functionalized TPA and carbazole derivatives to a triethoxysiloxane monomer via ureas. Homopolymers were prepared by the self-condensation of the corresponding hydrolyzed hydroxysiloxane monomer. The polymers consolidating pendant redox-active and emissive components were EFC in solutions, thin films, and in functioning EFC devices. **P4a** (Figure 13 and Figure 14B) showed the best performance, with a rapid fluorescence off/on response time of 1.2 s/3.5 s, and it had a high transmission ratio (83%). No significant decrease in the optical properties of the thin films was observed over 100 cycles at 10 s switching intervals. The intrinsic blue solid state of **P4b** could also be quenched electrochemically (Figure 14A). K. Su et al. designed a pendant pyrene polymer (**P5**) that was EFC in a thin film (Figure 14C) [139]. The polymer had a fluorescence contrast ratio of 83 and a fluorescence off/on response time of 2.8 s/0.9 s over 50 cycles of switching the applied potential.

Unconjugated copolymers containing TPA were also prepared by N. Sun et al. [140], who investigated the effect of structurally diverse polyimides with TPA incorporated in the alternating copolymer framework. The electroactive TPA was also rationally designed to be a constitutional component of the fluorene emitter. Their consolidated redox-active and fluorescent polyimides were prepared by condensing the diamino-TPA monomer with various complementary dianhydrides in stoichiometric amounts. The advantage of this design was that the amine of the TPA served as an electron-donating group that modulated the emission color of the emitter. The fluorene pendant polyimide (**P6**) had a moderate fluorescence yield of 28%. It had a contrast ratio upwards of 75 with only a 10% loss in the optical response after 300 cycles of switching the applied potential. The fluorescence could also be reversibly deactivated and activated with applied potentials of 1.2 and 0 V, respectively. With the aim of improving the EFC properties of unconjugated polymers, R. Zheng et al. evaluated the effect of varying the aromatic core in a polymer’s imide backbone [141]. The common components of their five polyimides (**P7**) were the pendant carbazole emitter and the TPA moiety that was integrated in the polyimide as a constitutional component. The redox potentials of the imides were consistent regardless of the imide aromatic core. The polymers were also consistently both electrochromic and electrofluorochromic and they had a similarly slow fluorescence off/on switching time of 10 s and 5.5 s. The optical contrast values of the polymers were 25% and 57%. All the polymers had good electrochemical stability with a maximum of 12% loss after 600 cycles of switching the applied potential. 

T. Yu et al. prepared **P8** polymers with varying degrees of freedom along the TPE polymer backbone [142]. The flexibility of the polymers affected the packing and the electrochemical robustness of the film. The more flexible **P8xc** had fewer defects and a better micromorphology according to SEM compared to **P8xa** after 500 cycles of switching the applied potential. In contrast, **P8xa** was the only polymer that was emissive in a thin film (Φ_fl_ = 15%) due to the AIE effect. It also presented good EFC performance with an only 18% decay in the fluorescence ratio over 300 cycles of potential switching in a half-device, maintaining an 80% fluorescence ratio. The advantage of the **P9** polymers that were developed by A. Bejan et al. [143] was their ambipolar redox activity. Therefore, they were anodically and cathodically active owing to their TPA and imide components, respectively, although only the anodic channel was reversible. Of the polymers examined, only **P9a** emitted in a functioning EFC device. The fluorescence ratio of the operating EFC device was 7.5 and it maintained 85% fluorescence recovery over 100 cycles of device operation. The fluorescence-bleaching and recovery times of the device were 6.4 s/9.7 s, respectively, and they were consistent in the half-devices. 

#### 5.1.2. Polyamides

**Figure 15:**
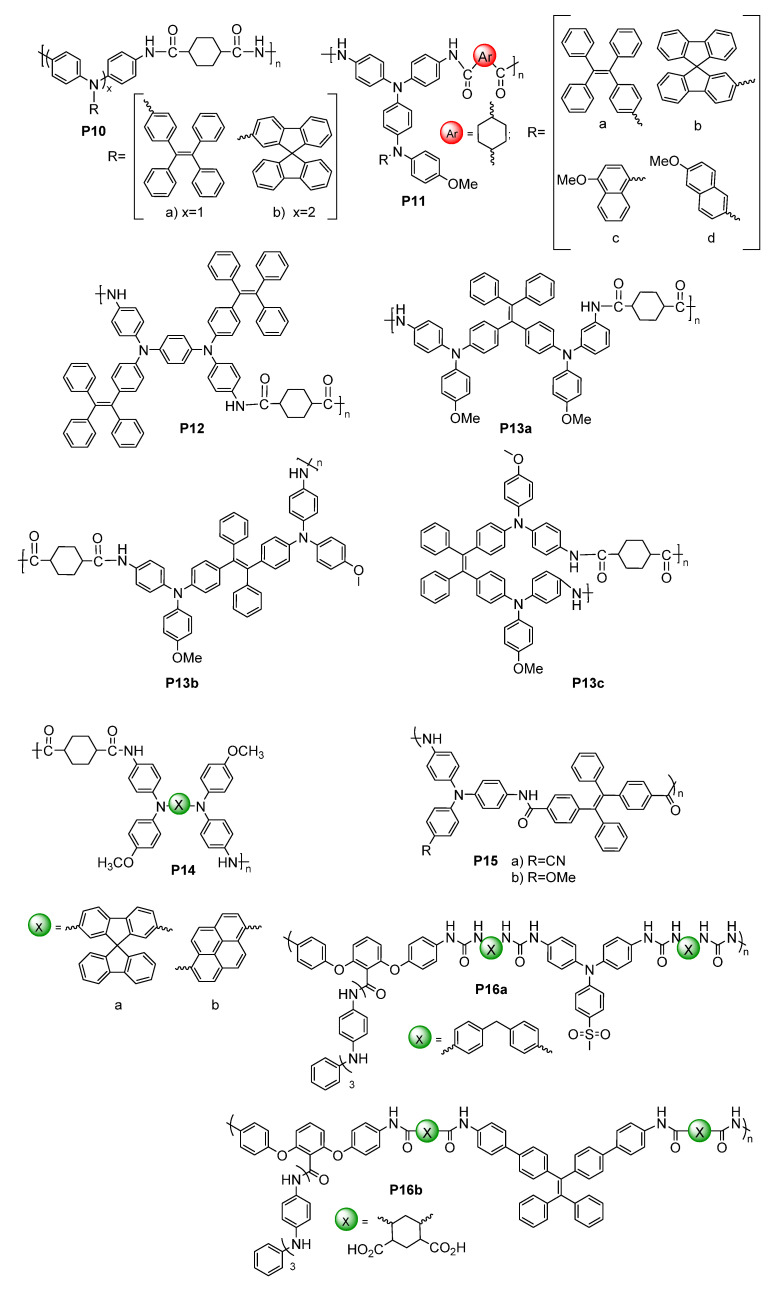
Polyamides with EFC properties [56,144,145,146,147,148,149,150,151,152,153].

Amides are alternative covalent linkages used to prepare unconjugated EFC polymers. These were exploited by N. Sun et al., who prepared **P10a** (Figure 15) consisting of the AIE-active TPE and TPA moieties [144]. The fluorescence-enhancing TPE was incorporated into the polymer as a pendant group by covalently coupling it with the redox-active TPA moiety. The latter comprised a polymer with alternating carbocycles via amide linkages. The polymeric films had a contrast ratio of 417 during 360 off/on cycles with off/on response times of 1.2 s and 6.7 s, respectively. The response time could be improved by making the film porous. This promoted the fast intercalation and ejection of the electrolyte in the polymer film during the switching of the potential. This aside, the electrofluorochromic stability of the polymer decreased after 150 cycles of switching the potential. 

A half-device of **P10b** was also investigated by X. Li et al. using a spirobifluorene as an emitter that was embedded into the TPA framework. The TPA segment was exploited as the redox-active component [153]. Despite its weak, blue solid-state emission (Φ_fl_ < 2%) owing to aggregation-induced quenching, the polymer was electrofluorochromic, with a moderate fluorescence contrast ratio of 111. The off/on responses times, namely, 1.2 and 4.2 s, respectively, were consistent with other polymers. The key performance characteristic of the 200–300 nm half-device of **P10b** was its consistent contrast, for which it lost < 11% after 800 cycles of switching the applied potentials at 20 s intervals. The film also showed a muted 50 nm solvatochromic shift in polar aprotic solvents.

**Figure 16:**
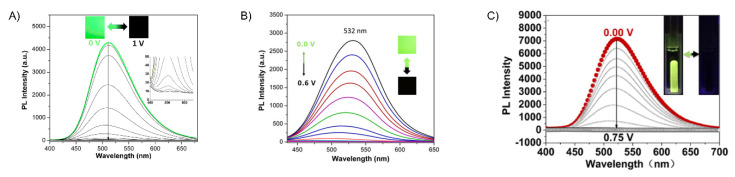
Electrofluorochromism of **P10a** (**A**), **P11a** (**B**), and **P13a** (**C**) in a half-device configuration [144,145,148]. Insets (**A**–**C**) photographs showing the deactivation/activation of fluorescence with applied potential. (**A**) Reproduced with permission from ref. [144]. Copyright 2018 American Chemical Society. (**B**) Reproduced with permission from ref. [145]. Copyright 2019 American Chemical Society. (**C**) Reproduced with permission from ref. [148]. Copyright 2020 American Chemical Society.

N. Sun et al. reported thin films of **P11a** (Figure 16B) with improved properties compared to **P10a** (Figure 16A) [145]. The electroactive TPA was both a repeating unit of the polyamide and a pendant group in **P11a**. The emitting TPE moiety was covalently attached to the pendant TPA. The fluorescence off/on response times of 0.6 s/4.4 s for **P11a** were faster than those of **P10a**, and it had a contrast ratio upwards of 82 at 20 s switching intervals. The films were stable over 300 switching cycles. Similarly, the spirobifluorenes were prepared to investigate the effect of incorporating the TPA moiety into the polymer (**P10b**) on the electrofluorochromic properties and compared to when it was added as a pendant group (**P11b**). K. Su et al. also assessed the effect of the redox inactive dicarboxycyclic repeating unit on the polymers’ emissive properties [146]. The alternating cyclohexyl polymer was the most fluorescent in solution of the two polymers (Φ_fl_ = 70%). Its emission was quenched five-fold when cast as a film (Φ_fl_ = 14%). The intrinsic emission of **P11b** in solution was comparable to its counterpart **P10b**. Unlike **P10b**, the cyclohexyl **P11b** retained its emission in a film, being nearly nine-fold greater than **P10b**. The cyclohexyl **P11b** exhibited an EFC behavior with a fluorescence contrast ratio of 67. It presented better performance in terms of response than its counterpart **P10b**, with a fluorescence off/on switching response of 0.3 s and 2.3 s, respectively, in a half-device. No degradation in the performance was observed after 200 cycles of fluorescence off/on switching. The spirobifluorene could be replaced with a naphthalene emitter without sacrificing the resulting device’s overall properties. The green-yellow emissive polymers (**P11c–d**) prepared by K. Su et al. maintained their solid-state emission, which was identical to **P10b**, but with a 30 nm emission red shift [56]. The fluorescence off/on response times of the half-devices were contingent on the naphthalene regioisomer, with **P11c** having response times similar to **P10b**. Both naphthalene polymers had a fluorescence contrast ratio upwards of 100 that decayed only by 10% after 500 cycles of switching the potential at 20 s intervals.

The polyamide **P12** was also prepared with two TPE pendant groups. This polymer fluoresced more in the solid state than **P12** in solution owing to its enhanced AIE effect afforded by the two pendant TPE moieties. The polymer’s contrast ratio was upwards of 252 in solution and 82 in a thin film, with off/on switching response times of 0.7 s and 2.1 s, respectively. The polymer was stable, presenting no significant decrease in the emission intensity even after 500 cycles of switching the potential at 20 s intervals [147]. The polymers were complemented with polyamides having both TPE and TPA incorporated into their polymer backbones. These polyamides differed in terms of the regiosubstitution of the TPE. The ethylene of the TPE was exo to the polymer backbone for **P13a** (Figure 15 and Figure 16c), whereas it adopted the *E* and *Z* isomers along the polymer backbone for **P13b** and **P13c**, respectively. All the polymers presented similar quasi-reversible oxidation concomitant with a colorless to black EC response. Of the three isomeric analogs, the *Z* isomer had the best overall properties. **P13c** had the fastest EC coloration and bleaching (0.6 s/0.3 s) and EFC off/on response (0.2 s/2.9 s). It also had good stability with a consistent performance over 300 switching cycles. Its EC contrast (92%) and EFC contrast ratio (838) were also higher than its isomeric counterparts [148]. K. Su et al. also exploited the amide linkages with two diphenylamines that were bridged either with a spirobifluorene (**P14a**) or pyrene (**P14b**) [149]. Diphenylamines, when coupled with aromatics, have similar redox activity and electrochemical reversibility to TPAs. Therefore, they can be reversibly switched between their neutral and their oxidized states, making them suitable for use in EC/EFC devices. The aromatic bridge between the two diphenylamines had little effect on the EC/EFC properties. Indeed, **P14a** and **P14b** had contrast ratios of 64% and 48% and rapid color-switching responses of 2.6 s/1.7 s and 2.8 s/2.4 s, respectively. The fluorescence contrast ratio of **P14a** and **P14b** was 300 and 210, respectively. **P14a** and **P14b** had rapid fluorescence off/on switching times of 1.3 s/1.4 s and 1.2 s/1.9 s, respectively. The performance of the polymers in operating EFC devices was consistent with a minimal loss of both the EC (<7.4%) and the EFC (<27%) ratios during 300 cycles of potential switching. Emission enhancement, contrast ratio improvement, and fast response times were enabled by the replacement of one of the phenyl substituents of the TPE with a nitrile. This was exploited by S. Yang et al., who covalently coupled TPA polymers with pendant triphenylacrylonitriles [52]. Their polyaniline-like films emitted upwards of 44% depending on the triphenylacrylonitrile substitution. The triphenylacrylonitriles also had an emission contrast ratio of ca. 4440 in working electrofluorochromic half-devices. The devices also had fast response times upwards of 180 ms.

The oxidation potential of TPA can be modified through substitution in its 4,4′,4″-positions. This was leveraged by S. Cheng et al., who prepared a series of TPA copolyamides substituted with either an electron acceptor nitrile or an electron-donating methoxy (**P15a–b**) [150]. The OMe substitution lowered the TPA oxidation potential and increased the PET quenching of the alternating TPE comonomer. In contrast, the nitrile group increased the oxidation potential and suppressed the level of PET emission quenching. The latter was evidenced by a three-fold increase in the Φ_fl_ of the nitrile polyamide relative to its methoxy counterpart. The fluorescence response times of the full devices of **P15a** and **P15b** were 7.1 and 6.5 s, respectively. The fluorescence contrast ratios were 64 and 48 for the nitrile and the methoxy polyamide, respectively, with 360 s cycles of switching the applied potential. The fluorescence contrast ratio decreased to ca. 10 when the applied potential was switched at 10 s intervals. Upwards of 94% of the original fluorescence could be recovered after 300 cycles of switching the applied potential.

The intrinsic emission of triphenylamine was exploited by Y. Yan et al. who prepared a polyurea copolymer of *p*-methylsulfonyl triphenylamine with an oligoaniline pendant group (**P16a**) [151]. Both the TPA and the oligoaniline components were electroactive, and they both underwent a color change. The copolymer fluoresced at 518 nm and its emission decreased by 3% upon the electrochemical oxidation of the oligoaniline pendant moiety. The photoinduced fluorometric off/on responses of the film in a half-device were 9.4 and 10.8 s, respectively, during 100 cycles of switching the applied potential. The off response accelerated to 6.2 s while the on response slowed to 14 s after 100 cycles of switching the applied potential. The TPA component of the polymer was then replaced with TPE (**P16b**) [152]. The AIE effect of the TPE was activated in a thin film, with the polymer fluorescing blue-green (Φ_fl_ = 14%) in a half-device. Upwards of 80% of the solid-state fluorescence could be quenched with an applied potential, although its fluorescence contrast ratio was low (ca. 8). The 90% off/on response time of the half-device was also slow, being 5.3/5.5 s. The contrast ratio was moderately consistent at 5.5 over 150 cycles of switching the potential at 15 s intervals. The fluorescence contrast ratio improved to 8 in an operating dual-display chromic device (Figure 19B). Materials that change both their color and photoemission intensity with an applied potential are termed dual-functional materials [142,149,154]. Their use as the active layer in devices results in dual-mode displays, with visually detectable color and photoemission changes with an applied potential [155,156]. **P16b** was electrospun into nanowires to develop a nanofibrous membrane for glucose detection. The EFC performance of the film was improved by replacing the cyclohexyldiamide polymer linkage with a hexyldiurea [157]. The half-device’s emission increased to 23%, and 97% of its emission could be quenched with an applied potential. While the fluorescence contrast ratio increased to 42, it decreased nearly four-fold during 200 cycles of potential switching. The off/on response time was also slower, doubling to 9.1/9.2 s. 

### 5.2. In Situ Polymerization of Electrofluorochromic Polymers

**Figure 17:**
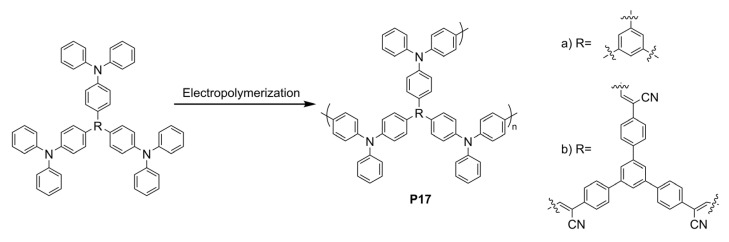
EFC polymer prepared by anodic electropolymerization [166].

Rather than synthesizing soluble polymers to be cast on electrodes to prepare EFC devices, polymers can be prepared directly on an electrode. This approach was widely used before the advent of advanced polymerization methods, which was in part due to its simplicity [158]. The advantage of using electropolymerization to prepare conjugated polymers consists of its relatively straightforward requirements: (i) the monomer has to be electroactive within the operating window of the electrolyte, and (ii) the resulting electrochemically generated intermediates must cross-couple. The major constraint of electropolymerization is the quantity of material that can be prepared, which is limited to a thin layer deposited on the working electrode. Nonetheless, this method continues to be used to prepare conjugated polymers [159,160,161,162], especially EC polymers [163], in order to establish structure–property relationships [164,165]. The preparation of EFC polymers has, however, been limited. The approach proceeds via the electrochemical generation of radical cations that self-couple. As their degree of conjugation increases, the polymers deposit on the working electrode, resulting in an insoluble, electroactive TPA film. TPA is a suitable monomer for such in situ electropolymerization. This approach was exploited by D. Santra et al. who prepared cross-linked films from a 1,3,5-TPA-substituted benzene starburst monomer via anodic polymerization (**P17a**; Figure 17) [166]. The film in a half-device had a fluorescence contrast ratio that was contingent on an acrylonitrile linker, varying between 64 (**P17a**) and 179 (**P17b**). The films of **P17a–b** maintained their contrast ratios during 100 cycles of potential switching, and the photoinduced emission of the dendrimers could be quenched in full devices. 

### 5.3. Electrofluorochromic Polymers Other Than TPA

**Figure 18:**
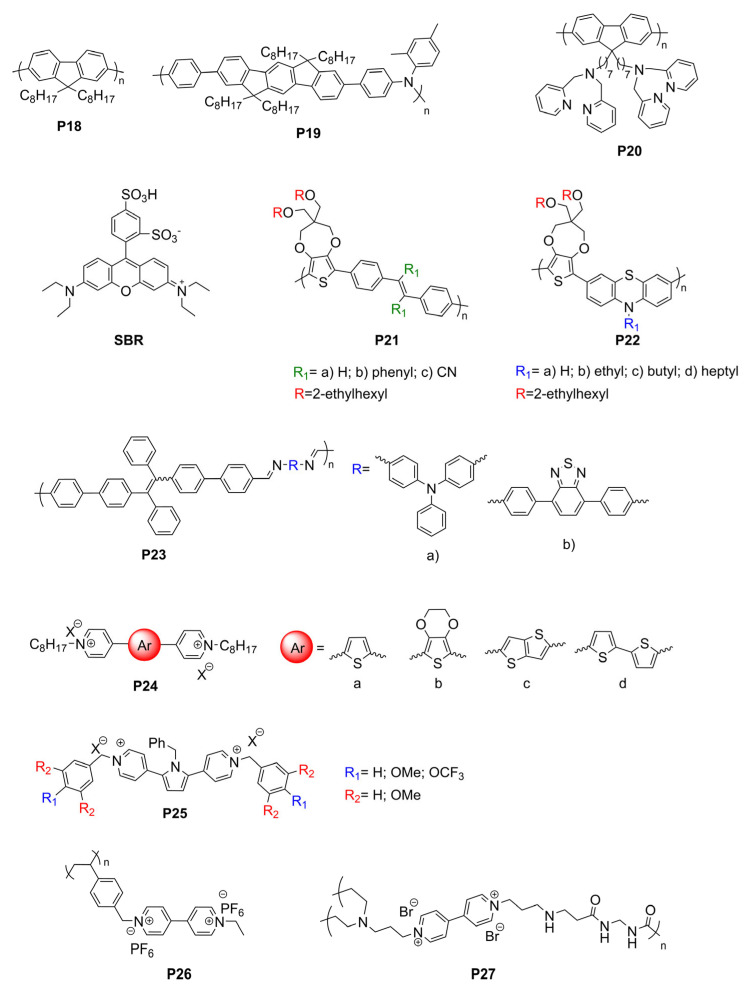
Recent conjugated and viologen-based EFC polymers [60,154,169,170,171,172,173,174,175].

Conjugated electrochromic polymers possess intrinsic properties that allow for their use as electrofluorochromes, namely, electroactivity and photoemission. Representative (yet limited) examples of conjugated polymers that can be used as electrofluorochromes are given in Figure 18. Fluorene is an intrinsic fluorophore that has been successfully used in emission applications [66,167,168]. It is also fluorescent when conjugated with other aromatics and it can be electrochemically oxidized. Polyfluorene can, therefore, potentially replace TPA for use in EFC devices and it can play a dual-role, both as the emitter and the redox-active segment. H. Lim et al. evaluated polyfluorene **P18** as an EFC polymer [169]. The fluorescence contrast ratio of the polyfluorene EFC device was 3.3 over 200 cycles at 10 s switching intervals of the applied potential. The extended polyfluorene **P19** was evaluated by M. Pietsch et al. in an operating EFC device that was fabricated by inkjet printing [154]. It had a fluorescence contrast ratio of 4 and an off/on response time of 4 s/3 s, respectively. The emission intensity decreased by more than 50% after 100 switching cycles at 40 s intervals. The limited stability when cycling between the applied potentials was assigned to the degradation of the transparent, conductive ITO on the device electrode and not because of a decrease in the polymer’s performance [154]. C. Xiang et al. designed polyfluorene **P20** with pendant pyridines [170]. The pendant pyridines intermolecularly interacted with the sulfonic groups of sulforhodamine B (SRB) to form a gel. The EFC measurements of the **P20**-**SBR** gel that did not use a supporting electrolyte showed no significant fluorescence quenching of the polymer. In addition, no performance degradation was observed during the limited 12 cycles of potential switching. 

J. Wu et al. prepared conjugated poly(phenylenevinylene) alternating copolymers by step-growth polymerization using either Suzuki or direct hetero(arylation) with an electron-rich ProDOT [174]. The latter was incorporated as the redox-active component to render the polymer electroactive. The authors examined the effect of vinylene substitution on the opto-electronic properties. Both the absorption and emission of the dinitrile polymer (**P21c**) were red-shifted relative to both its phenyl (**P21b**) and native (**P21a**) counterparts. Indeed, both the color and emission of **P21c** were orange, while **P21a–b** were colored yellow and they emitted green fluorescence. **P21a** and **P21c** exhibited dual-state emission, emitting both in solution and in the solid state, owing to multiple C—H⋯π bonds. These non-covalent interactions restricted intramolecular rotation whereas the TPE constitutional component of **P21b** promoted emission via the AIE effect. Regardless of the ethylene substitution, the set of polymers was electrofluorochromic, with their emission being reversibly quenched with an applied potential. Qualitatively, the emission of the nitrile polymer **P21c** was quenched to a greater degree than that of the other polymers. The fluorescent TPE in the ProDOT alternating conjugated copolymer could be replaced with another intrinsic emitter: phenothiazine. The **P22** polymers developed by J. Wu et al., with varying lengths of the *N*-alkyl substitution, were emissive in films, except **P22a** [171]. The length of the *N*-alkylation on the emitting polymers had a minimal effect on both the optical and the electrochemical properties. The emission of the green films could be reversibly quenched with an applied potential.

AIE-promoting TPE was also exploited by F. Liu et al. by incorporating an AIE emitter into the studied polymer (**P23**) [175]. Their approach relied on using imine bonds to form the polymer by condensing a dialdehyde TPA derivative with either a diamino TPA or diamino diphenylbenzothiadiazole. Qualitatively, the emission of the polymers was enhanced when aggregating in water/THF mixtures.

Viologens have been extensively used as the consolidated redox-active and color-changing component in operating EC devices [176,177]. This is due to their two reversible electrochemical reductions, which are both colored. Viologens can exhibit three colored states. The colors of viologens can be adjusted by modifying the counter anion, *N*-substitution, via structural modification, and by altering the applied voltage [178,179,180]. Viologens have not been considered as EFC materials because they are known fluorescence quenchers. Their efficiency of excited-state deactivation by PET is contingent on their charge. For instance, bipyridine is less efficient in deactivating the excited state than its dicationic viologen counterpart [181]. M. Chang et al. demonstrated that viologens (**P24**) could fluoresce by separating the two pyridine cations [173]. The Φ_fl_ of the viologens in solution increased from 2% for **P24a** to 72% for **P24d**, according to the distance separating the dications. Given that their intrinsic fluorescence can be controlled by structural modification concomitant with their intrinsic electroactivity and reversible electrochemical redox properties, viologens are emerging as viable EFC materials. The EFC behavior of viologens was further demonstrated in an electrolytic gel, with **P24c** having the highest fluorescence contrast ratio of 221. Functioning EFC devices fabricated from the viologens maintained their emission contrast ratio for 200 cycles of potential switching. The intrinsic emission of a pyrrole core could also be reversibly quenched by photoinduced electron transfer with the flanking viologens (**P25**). This was exploited by B. Deng et al. whose functioning EFC device had a consistent fluorescence contrast ratio of 600 over 50 cycles of switching the applied potential [182]. The emission of the viologens was highly dependent on the phenyl substituents. The single substitution (-OCF_3_) yielded the highest emission (34%), while the emission was quenched with the trisubstituted methoxy.

Fluorescence can also be imparted by making colloids with a viologen. This was taken advantage of by S. Qu et al. who covalently appended viologen to polystyrene (**P26**) [60]. A monolayer of the polystyrene-colloid with a pendant viologen was both redox-active and emissive. The emission of the colloids was quenched with an applied potential. It was established by fluorescence imaging that each reduced state (radical cation) could quench upwards of five fluorescent dications. The study showed that most of the viologen fluorescence could be quenched by electrochemically converting <10% of the dication to its reduced radical cation.

**Figure 19:**
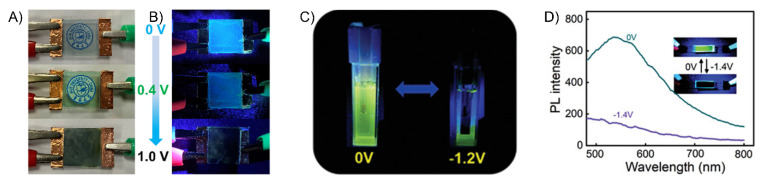
Operating dual-display device: electrochromic (**A**) and electrofluorochromic (**B**) devices developed from **P16b** with different applied potentials. Reproduced with permission from ref. [152]. Copyright 2021 Elsevier. (**C**) Photograph of **P27** in water irradiated at 410 nm with an applied potential of 0 V (left) and −1.2 V (right). (**D**) Fluorescence spectrum of dual-mode device of **P27** with an applied potential of 0 and −1.4 V and photographs of operating device (inset). (**C**,**D**) Reproduced with permission from ref. [172]. Copyright 2022 John Wiley and Sons.

To overcome viologens’ lack of emission, J. Zhao et al. exploited the autofluorescence of a hyperbranched polyamidoamine [172]. By copolymerizing a diamino viologen with *N*,*N*’-methylene bisacrylamide via a Michael addition, the otherwise non-fluorescent monomers yielded a hyperbranched polymer (**P27**) that emitted a green color (Figure 19C). The green emission could be quenched by electrochemically reducing the viologen to its radical cation. The fluorescence contrast of **P27** in solution was 8.5, and the fluorescence off/on times were 22.9/12.5 s. The switching times were improved to 6.6/3.2 s in an operating EFC device (Figure 19D) whose fluorescence contrast ratio did not degrade over 6000 s of device operation. These efforts show that viologens can have ideal EFC properties when their structures are judiciously engineered. 

### 5.4. Understanding Design Principles of Electrofluorochromic Polymers

Functioning EFC devices can be successfully prepared by integrating discrete molecular components, namely, a fluorophore and an electroactive moiety, into unconjugated polymers. Unlike their molecular counterparts in solution, solid-state emission is quintessential for the operation of EFC devices. High Φ_fl_ is also a key performance indictor of EFC polymers. Both of these criteria can be met by integrating dual-emission materials into the polymer (**P4**, **P10a**, and **P13–15**). The color of the perceived emission can be extended across the visible region by conjugating the dual-emission segment with various aromatics that have different degrees of electron richness (**P8**, **P21**, and **P22**; Table 2). By adopting these design principles, sub-second fluorescence off/off rates can be achieved in EFC devices (**P21**), along with desirable high-fluorescence contrast ratios. Unconjugated polymers consisting of an intrinsic solid-state emitter and an electroactive constitutional component can also operate as EC devices. Such dual-display devices can both change their color and their emission with applied potential, thus expanding their range of applications. Like their molecular counterparts, the cathodic channel of the unconjugated electrofluorochromic polymers can be accessed within the electrochemical operating window of the device by incorporating electron-deficient heterocycles (**P24–27**). The device performance, including short coloration/bleaching times and the ability to withstand an extended number of switching cycles of an applied potential, can be enhanced with improvements to the quality of the polymer film. Towards this end, porous films improve the response times of EFC devices by favoring electrolyte intercalation into the polymer film (**P10A**). Overall, the device performance is underpinned by both the intrinsic redox and emission properties of the polymer’s constitutional components, along with the interfacial contacts, morphology, and thickness of the film, among other parameters in the assembled EFC device. 

## 6. Covalent Organic Frameworks

Covalent organic frameworks (COFs) are persistent 2D or 3D networks whose structure is maintained by covalent bonds [183]. This contrasts with its metal–organic framework counterparts that are reliant on metal–ligand bonds to support their ordered networks [184]. Both the shape and size of the networks of COFs can be adjusted depending on the constitutional components used for their preparation. As a result, their pore sizes, dimensionality, the length of their channels, and the size of their persistent structures can be modulated [185,186]. The properties of COFs, and, ultimately, their application, can also be tailored depending on the intrinsic properties of the constitutional building units [187]. Furthermore, COFs have the advantage of being crystalline unlike their polymer counterparts, which are amorphous [188].

**Figure 20:**
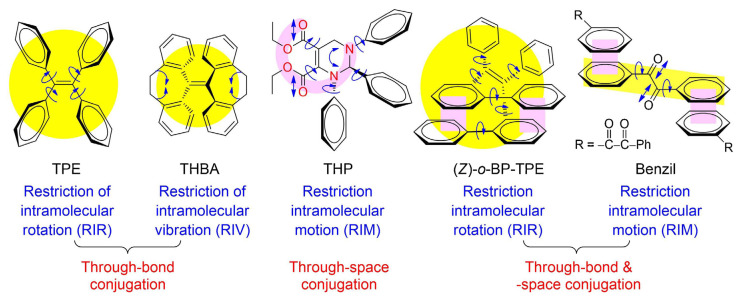
Different restricted motions that can be leveraged to promote AIE in COFs [189]. Reproduced with permission from ref. [189]. Copyright 2015 Elsevier.

COFs can emit via different deactivation mechanisms (ICT, FRET, and PET) when they are excited by a stimulus (light, heat, and/or electricity) [189]. Their emission is highly dependent on rotational, vibrational, and intramolecular motions (Figure 20). These emission deactivation modes can be attenuated to produce fluorescent COFs by casting the frameworks either from a poor solvent or by forming films. The fluorescence-quenching motions can be further restricted by incorporating the deactivating moiety next to a conjugated bond.

**Figure 21:**
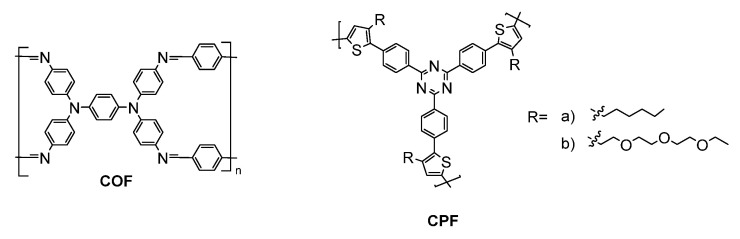
Representative electroactive covalent organic frameworks **COF** and **CPF** [192,194,196].

The chemical robustness of COFs combined with their opto-electronic properties make them ideal electrochromes and electrofluorochromes [190,191,192,193]. Indeed, electrochromic COFs have been prepared from tetra amino TPA and terephthalic dialdehyde. The resulting **COF** (Figure 21), produced by Q. Hao et al., had three colored states with an applied potential: orange at −0.2 V, grey at 0.75 V, and blue at 0.9 V [194]. The color contrast of the **COF** was ca. 55% at both 740 and 1050 nm, with no degradation of the contrast during 20 switching cycles. COFs exhibiting similar EC device performance were also prepared from diaminoanthraquinone and triformylresorcinol, using functionalized graphene oxide to enhance their conductivity [195]. S. Halder et al. also prepared electrofluorochromic, conjugated polymeric frameworks (**CPF**; Figure 21) via the Suzuki coupling of trisphenyltriazine boronic acid with dibromothiophene [196]. The frameworks were both electrochromic and electrofluorochromic. The **CPFa** and **CPFb** films presented blue-green emission at 515 and 524 nm, respectively. This emission was reversibly and completely quenched upon electrochemical oxidation. No apparent degradation in the fluorescence contrast ratio was observed during five cycles of switching the potential. To benchmark the CPF performance, porous films were compared to their linear counterpart, which was not EFC. In fact, the inherent porosity of the CPF promoted faster ion diffusion kinetics, resulting in improved EC and EFC performance.

## 7. Perspective and Future Outlook

COFs meet the key emission [197] and redox activity [193,198] requirements with which to be effectively electrofluorochromic. In addition, their intrinsic porosity is ideal for electrolyte migration. This reduces their response times with an applied potential and potentially allows them to switch faster than their polymer counterparts. An additional benefit of COFs is their structural diversity, which provides the means to tune both their emission color and redox potential by modifying their structure. Leveraging the fluorescence-enhancing design rules, it would be possible to design highly emissive COFs for electrofluorochromic applications [189,199]. The straightforward preparation of COFs and their properties that can be tuned by structural modification collectively position COFs to become the next generation of high-performance EFC materials.

Electrofluorochromism has been widely explored over the past five years. Many molecular and polymeric materials have been investigated as suitable EFC materials whose photoinduced fluorescence intensity can be modulated with an applied potential. While molecular fluorophores are advantageous due to the capacity for their properties to be tailored, including with respect to their intrinsic high-emission yields via structural modification by synthetic means, their polymeric counterparts are more resilient towards multiple redox cycles. This is a key operating device performance metric. Nonetheless, structure–property relationships can be accurately established with molecular electrofluorochromes and these have helped understand the molecular requirements for electroactivity and intrinsic solid-state fluorescence. Toward this end, TPA continues to play an important role in providing critical, reversible electroactivity that is required for the electrochemical modulation of photoinduced emission intensity because of its reversible electrochemical oxidation and emission. Meanwhile, AIE and the design criteria for achieving solid-state emission are opening new channels for the rational design of EFC materials. The availability of dual-state emitters though synthetic methods and their covalent coupling with redox-active moieties are becoming standard means of producing EFC materials. Moreover, tuning the emission wavelength by structurally modifying the AIE emitter opens the possibility of developing EFC materials that rival their EC counterparts, providing a palette of emission colors that can potentially cover the visible spectrum and extend into the NIR region. The incorporation of these functional moieties into polymers constitutes a viable avenue through which to increase the long-term performance of EFC devices, especially their lifetime, and to provide increased electrochemical robustness and consistent photoemission contrast ratios during prolonged device operation. An advantage of polymer electrofluorochromes over their molecular counterparts is their tailorable mechanical properties. Ideal properties would be compliant with flexible substrates, creating the opportunity for truly stretchable, bendable, and flexible operating EFC devices. An additional advantage of electrofluorochromic materials is that they are also EC. Therefore, they can play two roles in dual-display devices, namely, enabling color-switching and fluorescence intensity modulation. This expands the uses of EFC devices beyond those of conventional EC devices. While the molecular design principles for creating EFC materials are becoming well established, overall EFC device performance remains underpinned by electrochemical and photophysical processes that take place in each layer and at the interface of the different device layers. Understanding the interplay of these solid-state processes with the layer thickness, the electrolyte, and the electrode composition will be key for developing EFC devices with long-term stability and consistent performance during their extended lifetime. The complex interplay of redox activity and the photoemission of the resting state can be simplified by exploiting the intrinsic emission of the electrochemically generated state without relying on the emission of the resting state. The next generation of high-performance EFC devices will undoubtedly integrate either permanent 2D or 3D structures to control porosity. This will promote electrolyte permeability and reversible electrolyte intercalation into the EFC material without affecting its ordered structure, resulting in a rapid photoemission response with an applied potential.

## Figures and Tables

**Table 1:** Collective spectroscopic and electrochemical data of molecular electrofluorochromes.

Cmpd	λ_abs_^a^ (nm)	λ_em_^a^ (nm)	E_ox_^b^ (V)	E_red_^b^ (V)	Φ_fl_ (%) ^c^	Cmpd	λ_abs_^a^ (nm)	λ_em_^a^ (nm)	E_ox_^b^ (V)	E_red_^b^ (V)	Φ_fl_ (%) ^c^
**AD1**	390, 410, 440 ^d^	650 ^d^	1.28 [1.24] ^d,p^	−0.96 [−1.00] ^d,p^	4 ^d,u^	**F5**	360 ^f^	442 ^f^	0.41 [0.37] ^o,p^		3 ^v^ (2.2)
**AD2a**	475 ^d^	688 ^d^	3.30^t^		(4)	**F6**	316 ^f^	471 ^f^	0.53 [0.49] ^o,p^		2 ^v^ (30)
**AD2b**	475 ^d^	628 ^d^	3.10 ^t^		(35)	**F7**	415 ^f^	596 ^f^	1.04 [1.00] ^n,p^		<1 ^v^ (44)
**AD3**	289 ^e^	471 ^e^	3.20 ^t^		27 ^d^	**F8**	423 ^f^	599 ^f^	1.05 [1.01] ^n,p^		<1 ^v^ (74)
**MV1**	486 ^d^	610 ^d^	0.71 [0.67] ^d,p^	−1.11 [−1.15] ^d,p^		**F9**	566 ^k^	593 ^k^		[−1.9, −1.15] ^m,s^	100 ^k^
**MV2**	240, 310, 375 ^e^	426 ^e^	0.71, 0.85 [0.67, 0.81] ^l,p^		(63)	**F10**	580 ^k^	614 ^k^		[−1.71, −0.91] ^m,s^	89 ^k^
**MV3**	378 ^f^	422 ^f^	0.53, 0.87 [0.57, 0.91] ^h,p^		70 ^f^	**F11**	522 ^h^	414 ^h^		−0.95 [−0.99] ^f,p^	
**MV4**	380 ^f^	496 ^f^	0.80 [0.76] ^h,p^		83 ^f^	**F12a**	470, 512 ^j^	430 ^j^		−0.89 [−0.93] ^f,p^	66 ^k^
**MV5**	397 ^f^	535 ^f^	0.86 [0.82] ^h,p^		23 ^f^	**F12b**	482, 526 ^j^	455 ^j^		−0.98 [−1.02] ^f,p^	61 ^k^
**MV6**	345 ^f^	441 ^f^	0.73 [0.69] ^h,p^		67 ^f^	**F12c**	496, 582 ^j^			−0.71 [−0.75] ^f,p^	
**MV7**	355 ^f^	496 ^f^	0.81 [0.77] ^h,p^		75 ^f^	**F12d**	502, 608 ^j^			−0.65 [−0.69]^f,p^	
**MV8**	368 ^f^	524 ^f^	0.78 [0.74] ^h,p^		52 ^f^	**F12e**	466, 504, 572 ^j^			−0.72 [−0.76] ^f,p^	
**MV9**	485 ^g^	615 ^g^	0.80 [0.42] ^h,p^		71 ^g^	**F12f**	406, 556 ^j^	445 ^j^		−0.92 [−0.96] ^f,p^	58 ^w^
**MV10**	527 ^d^	706 ^d^	0.79 [0.75] ^h,p^		3.4 ^d^	**F13a**	348 ^j^	420 ^j^	1.62 [1.58] ^j,p^	−1.49, −1.77, −2.50 [−1.53, −1.81, −2.54] ^j,p^	14 ^j^
**MV11a**	459 ^d^	621 ^d^	0.99 [0.95] ^h,p^		63 ^d^	**F13b**	356 ^j^	434 ^j^	1.66 [1.62] ^j,p^	−1.37, −1.59, −2.37 [−1.41, −1.63, −2.41] ^j,p^	24 ^j^
**MV11b**	477 ^d^	649 ^d^	1.19 [1.15] ^h,p^		47 ^d^	**F14a**	352 ^j^	385 ^j^		[−1.44] ^j,r^	73 ^j^
**MV11c**	460 ^d^	630 ^d^	0.92 [0.88] ^h,p^		72 ^d^	**F14b**	359 ^j^	395 ^j^		[−1.15, −1.55] ^j,r^	66 ^j^
**MV12**	400 ^h^	460 ^h^	[0.97] ^h,s^		30 ^h^	**F14c**	367 ^j^	410 ^j^		[−0.91, −1.32] ^j,r^	42 ^j^
**MV13**		674 ^e^	0.28 [0.36] ^h,q^	−1.78, −1.30 [−1.70, −1.22] ^h,q^	77 ^e^	**F14d**	375 ^j^	410 ^j^		[−0.75, −1.10] ^j,r^	30 ^j^
**MV14**	390 ^i^	452 ^i^		[−0.57, −0.53] ^i,s^	87 ^u^	**F15a**	345 ^l^ (364)	434 ^l^ (462)	[1.11] ^n,p^		<1 ^v^ (98)
**Pyr**	590–630	620–675	[0.53–0.73] ^h,q^		(5–11)	**F15b**	355 ^l^ (372)	451 ^l^ (478)	[0.79] ^n,p^		<1 ^v^ (91)
**F1**	380, 445 ^j^	498 ^j^		−0.13, −1.11 [−0.53, −1.51] ^m,q^	59 ^j^	**F16a**	380 ^l^ (413)	581 ^l^ (531)	1.21 [1.17] ^n,p^		4 ^v^ (25)
**F2**		(560)		−0.48 [−0.52] ^h,p^		**F16b**	400 ^l^ (430)	636 ^l^ (560)	0.99 [0.95] ^n,p^		<1 ^v^ (48)
**F3**	350 ^d^	510 ^d^	0.56 [0.10] ^h,q^		(6)	**F17**	522 ^h^	735 ^h^	[0.91, 1.19] ^h,r^	[−1.14, −1.41] ^h,r^	7 ^h^
**F4**	380 ^d^	500 ^d^	0.61 [0.15] ^h,q^		(3)	**F18**	288 (396) ^i^	(545)		[−3.23] ^t^	

^a^ Measured in given solvent. Parentheses—measured in solid state; brackets—measured in operating device. ^b^ Cyclic voltametric data converted to SCE from reported data. Brackets: reported cyclic voltametric data vs. given reference. ^c^ Fluorescence quantum yield measured with an integrating sphere in solution. Parentheses: measured in solid state. Measured in given solvent: ^d^ THF, ^e^ Toluene, ^f^ DMSO, ^g^ Hexane, ^h^ Dichloromethane, ^i^ H_2_O, ^j^ DMF, ^k^ MeOH, ^l^ NMP, ^m^ Acetonitrile, ^n^ Propylene carbonate, and ^o^ γ-Butyrolactone. Reported reference for cyclic voltametric data: ^p^ Silver/silver chloride, ^q^ Ferrocene/ferrocenium, ^r^ Silver/silver^+^, ^s^ SCE, ^t^ Potential applied in functioning device to induce fluorescence quenching. ^u^ Relative to 9,10-diphenylanthracene (Φ_fl_ = 100%). ^v^ Relative to quinine sulfate at 25 °C (10 μM in 1 N H_2_SO_4_; Φ_fl_ = 54.6%). ^w^ Relative to coumarin 153 in acetonitrile.

**Table 2:** Collective spectroscopic and electrochemical data of electrofluorochromic polymers.

Cmpd	λ_abs_ (nm) ^a^	λ_em_ (nm) ^a^	E (V) ^b,c^	Φ_fl_ (%) ^d^	τ_c_ (s) ^e^	τ_b_ (s) ^f^	Cmpd	λ_abs_ (nm) ^a^	λ_em_ (nm) ^a^	E (V) ^b,c^	Φ_fl_ (%) ^d^	τ_c_ (s) ^e^	τ_b_ (s) ^f^
**P1a**	360 ^g^	495 ^g^	0.84, 1.55 [0.80, 1.51] ^g,s^	69 ^g^	1.6	2.7	**P11c**	332 (327) ^g^	528 (486) ^g^	1.08, 0.66 [1.04, 0.62] ^h,s^	26.6 (15.2) ^g^	1.8	1.2
**P1b**	359 ^g^	495 ^g^	1.0, 1.4 [0.96, 1.36] ^g,s^	79 ^g^	4.2	3.6	**P11d**	332 (327) ^g^	512 (470) ^g^	1.07, 0.70 [1.03, 0.66] ^h,s^	83.8 (18.8) ^g^	2.2	1.1
**P2a**	346 (340) ^i^	455 (450) ^i^	0.70, 1.06 [0.66, 1.02] ^h,s^	26 ^t^	3.4	2.8	**P12**	316 (342) ^g^	525 ^g^	0.78, 1.16 [0.74, 1.12] ^h,s^		1.3	0.5
**P2b**	375 (378) ^i^	520 (491) ^i^	0.81, 1.11 [0.77, 1.07] ^h,s^	17 ^t^	2.2	2.1	**P13a**	312 (310) ^g^	532 (520) ^g^	1.08 [1.04] ^h,s^	(16)	2.2	0.8
**P2c**	356 (346) ^i^	505 (493) ^i^	0.87, 1.26 [0.83, 1.22] ^h,s^	4 ^t^	3.6	3.8	**P13b**	312 (310) ^g^	546 (523) ^g^	0.87 [0.83] ^h,s^	(25)	1.1	0.6
**P3a**	373 (370) ^g^	530 (515) ^g^	0.51, 0.79 [0.81, 1.09] ^h,s^				**P13c**	315 (312) ^g^	547 (526) ^g^	0.87 [0.83] ^h,s^	(33)	0.6	0.3
**P3b**	373 (370) ^g^	535 (521) ^g^	0.46, 0.69 [0.76, 0.99] ^h,s^	87 ^g^	0.7	5.6	**P14a**	312 (322) ^g^	432 (440) ^g^	0.76, 0.97 [0.72, 0.93] ^h,s^	54 ^g^ (8.4)	2.7	1.7
**P3c**	374 (369) ^g^	541 (532) ^g^	0.32, 0.65 [0.62, 0.95] ^h,s^				**P14b**	309 (309) ^g^	553 (530) ^g^	1.02 [0.98] ^h,s^	86 ^g^ (14)	2.8	2.4
**P4a**	303 (334) ^g^	401 (407) ^g^	0.36 [0.32] ^h,s^	18 (13) ^g,t^	1.2	3. 5	**P15a**	339 (343) ^g^	493 (510) ^g^	[2.10] ^p^	3 ^g,t^ (16)		
**P4b**	348 (348) ^g^	379 (380) ^g^	1.07 [1.03] ^h,s^	32 (30) ^g,t^	1.9	4.4	**P15b**	347 (353) ^g^	498 (554) ^g^	[1.75] ^p^	<1 ^g,t^ (5)		
**P4c**	304 (351) ^g^	398 (400) ^g^	0.48 [0.44]^h,s^	17 (13) ^g,t^	1.0	2.5	**P16a**	330 ^h^	515 (566) ^h^	1.40, 1.00 [1.36, 0.96] ^h,s^		2.6, 12.1, 6.7	2.2, 6.5, 3.6
**P4d**	315 (332) ^g^	479 (450) ^g^	0.65 [0.61] ^h,s^	37 (33) ^g,t^	1.4	3.6	**P16b**	365 ^j^	490 ^j^	0.55 [0.51] ^j,s^	14 ^t^	1.9	11.6
**P5**	332 (334) ^g^	541 (512) ^g^	0.59 [0.55] ^h,s^	49 ^g^	0.9	2.8	**P17**	342 (356)	412 (473) ^g^	[0.76, 1.02] ^h,q^		1.1	0.8
**P6**	329 (336) ^g^	400 (414) ^g^	1.47 [1.43] ^h,s^	28	7.3	1.5	**P18**	/	428 ^p^	1.29 [1.25] ^k,s^			
**P7a**	326 (267) ^g^	435 (450) ^g^	0.86, 1.11 [0.82, 1.07] ^h,s^	14 ^t^	10	7.7	**P19**	395 ^g^	440 (465) ^g^	[0.45, 0.90] ^h,r^			
**P7b**	326 (284) ^g^	382 (401) ^g^	0.85, 1.07 [0.81, 1.03] ^h,s^	23 ^t^	7.5	6.0	**P20**	376 ^o^	440 (577) ^o^	0.89, −1.41 [0.85 (−1.45)] ^o,s^			
**P7c**	324 (259) ^g^	399 (408) ^g^	0.84, 1.08 [0.80, 1.04] ^h,s^	46 ^t^	9.0	7.5	**P21a**	441 (452) ^n^	533 (452) ^n^	[1.37] ^h,q^		0.8	3.7
**P7d**	327 (274) ^g^	453 (487) ^g^	0.86, 1.01 [0.82, 0.97] ^h,s^	27 ^t^	8.5	5.5	**P21b**	402 (412) ^n^	538 (540) ^n^	[1.69] ^h,q^		4.0	2.0
**P7e**	324 (284) ^g^	395 (410) ^g^	0.92, 1.14 [0.88, 1.10] ^h,s^	6 ^t^	10	8.0	**P21c**	480 (522) ^n^	617 (636) ^n^	[1.32] ^h,q^		0.2	2.5
**P8xa**	310 (350) ^g^	535 (525) ^g^	0.47, 0.51 [0.43, 0.47] ^h,s^	<1 (15) ^g^			**P22a**	402 (420) ^l^	511 ^l^	[0.95] ^h,q^		0.4	2.1
**P8ya**	321 (325) ^g^	544 (534) ^g^	0.60 [0.56] ^h,s^	<1 (<1) ^g^	2.1	1.7	**P22b**	422 (445) ^l^	512 (534) ^l^	[1.17, 0.80] ^h,q^		0.6	1.5
**P8xb**	314 (348) ^g^	525 (500) ^g^	0.64, 1.10 [0.60, 1.06] ^h,s^	<1 (1) ^g^			**P22c**	424 (446) ^l^	513 (535) ^l^	[1.06, 0.79] ^h,q^		0.5	1.0
**P8yb**	325 (366) ^g^	562 (577) ^g^	0.70 [0.66] ^h,s^		2.5	1.5	**P22d**	420 (446) ^l^	512 (541) ^l^	[1.31, 0.82] ^h,q^		0.4	2.6
**P8xc**	300 (344) ^g^	465 (460) ^g^	0.55, 0.85 [0.51, 0.81] ^h,s^	<1 (3) ^g^			**P23a**	304 (325) ^m^		1.33 [1.29] ^h,s^		6.45	1.07
**P8yc**	318 (354) ^g^	525 (354) ^g^	0.65 [0.61] ^h,s^	(<1)	10.7	6.8	**P23b**	365 (362) ^m^		1.44 [1.40] ^h,s^		3.43	1.41
**P9a**	322 ^p^	399 ^l^	[0.91] ^k,q^		5.4	5.4	**P24a**	376 ^g^	433 ^g^	[−0.41, −0.17] ^g,q^	2 ^g^	0.9	24.8
**P9b**	463 ^p^		[0.90] ^k,q^		12.8	13.6	**P24b**	416 ^g^	480 ^g^	[−0.53, −0.30] ^g,q^	5 ^g^	1.9	21.6
**P9c**	384 ^p^		[0.92] ^k,q^		14.0	15.7	**P24c**	423 ^g^	466 ^g^	[−0.44] ^g,q^	16 ^g^	5.7	8.5
**P10a**	315 (441) ^g^	511 (514) ^g^	1.12 [1.08] ^h,s^	2 ^g^ (69)			**P24d**	437 ^g^	507 ^g^	[−0.33] ^g,q^	72 ^g^	2.9	35.1
**P10b**	349 (351) ^g^	480 (465) ^g^	1.11, 0.74 [1.07, 0.70] ^h,s^	44 (2) ^g^	2.9	1.8	**P25**	(410)	(465)	−1.1–−1.19; −1.31–−1.37 ^s^	<1–34	18–38	27–49
**P11a**	333 (334) ^g^	532 ^g^	0.66, 1.02 [0.62, 0.98] ^h,s^	<1 ^g^ (29)	2.0, 1.6	1.7, 1.2	**P26**	488 ^p^	570 ^p^	[−0.32] ^p,s^			
**P11b**	331 (329) ^g^	497 (456) ^g^	0.72, 1.11 [0.68, 1.07] ^h,s^	70 ^g^ (14)	2.3	1.6	**P27**	445 ^j^	530 ^j^	−0.87, −0.48 [−0.91, −0.52] ^j,s^		7.5	6.8

^a^ Measured in given solvent. Parentheses: measured in solid state. ^b^ Cyclic voltametric data converted to SCE. Brackets: reported cyclic voltametric data. ^c^ Positive values are electrochemical oxidations, negative values are electrochemical reductions. ^d^ Fluorescence quantum yield measured with an integrating sphere in solution. Parentheses: measured in solid state. ^e^ Time required to reach 90% transmittance of colored state. ^f^ Time required to reach 90% transmittance of bleached state. Measured in given solvent: ^g^ NMP, ^h^ Acetonitrile, ^i^ Dichloromethane, ^j^ H_2_O, ^k^ Propylene carbonate, ^l^ Chloroform, ^m^ DMF, ^n^ THF, ^o^ Tetrachloroethane. ^p^ Functioning device. Reported cyclic voltametric reference: ^q^ Silver, ^r^ Ferrocene/ferrocenium, ^s^ Silver/silver chloride. ^t^ Relative to quinine sulfate at 25 °C (10 μM in 1 N H_2_SO_4_, Φ_fl_ = 54.6%).

## Data Availability

Not applicable.
